# Long Intergenic Noncoding RNAs Affect Biological Pathways Underlying Autoimmune and Neurodegenerative Disorders

**DOI:** 10.1007/s12035-022-02941-0

**Published:** 2022-07-07

**Authors:** Patrycja Plewka, Katarzyna Dorota Raczynska

**Affiliations:** 1 Department of Gene Expression, Institute of Molecular Biology and Biotechnology, Faculty of Biology, Adam Mickiewicz University, Poznan, Poland; 2 Center for Advanced Technology, Adam Mickiewicz University, Uniwersytetu Poznanskiego 10, 61-614 Poznan, Poland

**Keywords:** Long intergenic noncoding RNAs, Neurodegenerative diseases, Autoimmune diseases, Multiple sclerosis, Alzheimer’s disease, Parkinson’s disease

## Abstract

Long intergenic noncoding RNAs (lincRNAs) are a class of independently transcribed molecules longer than 200 nucleotides that do not overlap known protein-coding genes. LincRNAs have diverse roles in gene expression and participate in a spectrum of biological processes. Dysregulation of lincRNA expression can abrogate cellular homeostasis, cell differentiation, and development and can also deregulate the immune and nervous systems. A growing body of literature indicates their important and multifaceted roles in the pathogenesis of several different diseases. Furthermore, certain lincRNAs can be considered potential therapeutic targets and valuable diagnostic or prognostic biomarkers capable of predicting the onset of a disease, its degree of activity, or the progression phase. In this review, we discuss possible mechanisms and molecular functions of lincRNAs in the pathogenesis of selected autoimmune and neurodegenerative disorders: multiple sclerosis, rheumatoid arthritis, systemic lupus erythematosus, Sjögren’s syndrome, Huntington’s disease, Parkinson’s disease, Alzheimer’s disease, and amyotrophic lateral sclerosis. This summary can provide new ideas for future research, diagnosis, and treatment of these highly prevalent and devastating diseases.

**Supplementary Information:**

The online version contains supplementary material available at 10.1007/s12035-022-02941-0.

## Introduction

Only 2–3% of the mammalian genome is transcribed into protein-coding mRNAs, and ~ 80% of the human genome exhibits some type of biochemical activity, such as RNA transcription, binding of transcription factors, or chromatin structure and histone modifications, in at least one cell type. Transcripts derived from these noncoding regions do not possess any protein-coding capacity and are annotated as noncoding RNAs (ncRNAs) [1]. Noncoding transcripts longer than 200 nucleotides are called long noncoding RNAs (lncRNAs). Long intergenic noncoding RNAs (lincRNAs) account for almost half of lncRNAs, and their genes are located from several bases to more than 3 Mb away from the nearest protein-coding gene. Similar to mRNAs, they are transcribed by RNAP2 (RNA polymerase II) and can be spliced, capped, and polyadenylated. However, in comparison to mRNAs, lincRNAs are expressed at a tenfold lower level, enriched in the nucleus, and mainly expressed in a cell type-specific, tissue-specific, developmental stage-specific, or disease state-specific manner, with overrepresentation in the brain and testis [2]. In humans, lincRNAs have diverse roles in gene expression and participate in a spectrum of biological processes. Within the nucleus, lincRNAs occupy the chromatin fraction and are involved in epigenetic regulation. They can affect transcription by recruiting chromatin remodeling complexes and forming R-loop structures at promoter regions. They can act as decoys to sequester transcription factors from their genomic targets or as scaffolds to enable the formation of multiprotein complexes and the recruitment of RNAP2. LincRNA loci can function *in trans*, producing lincRNA transcripts that function at locations genetically unlinked and spatially distant from their site of production. Other lincRNA loci regulate gene expression *in cis*, exhibiting transcriptional enhancer-like activity toward neighboring genes. Within the cytoplasm, they play a role in the modulation of mRNA stability, translation, and posttranslational modifications, as well as the regulation of their RNA interacting partners, e.g., miRNAs [2–7].

**Fig. 1:**
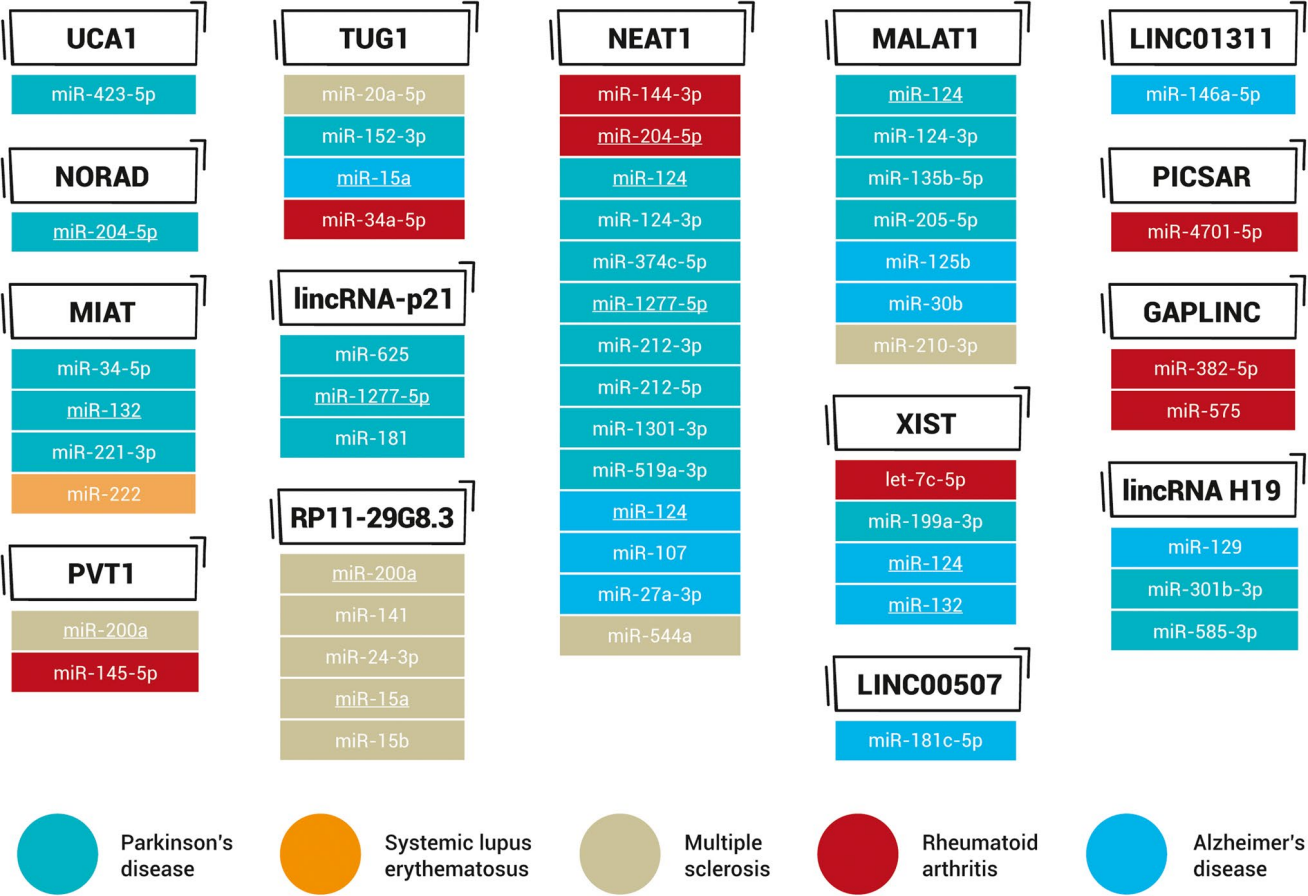
LincRNAs acting as miRNA sponges in the pathogenesis of autoimmune and neurodegenerative diseases. Colors refer to the lincRNA/miRNA axes related to a particular disease. Repeating miRNAs are underlined

**Fig. 2:**
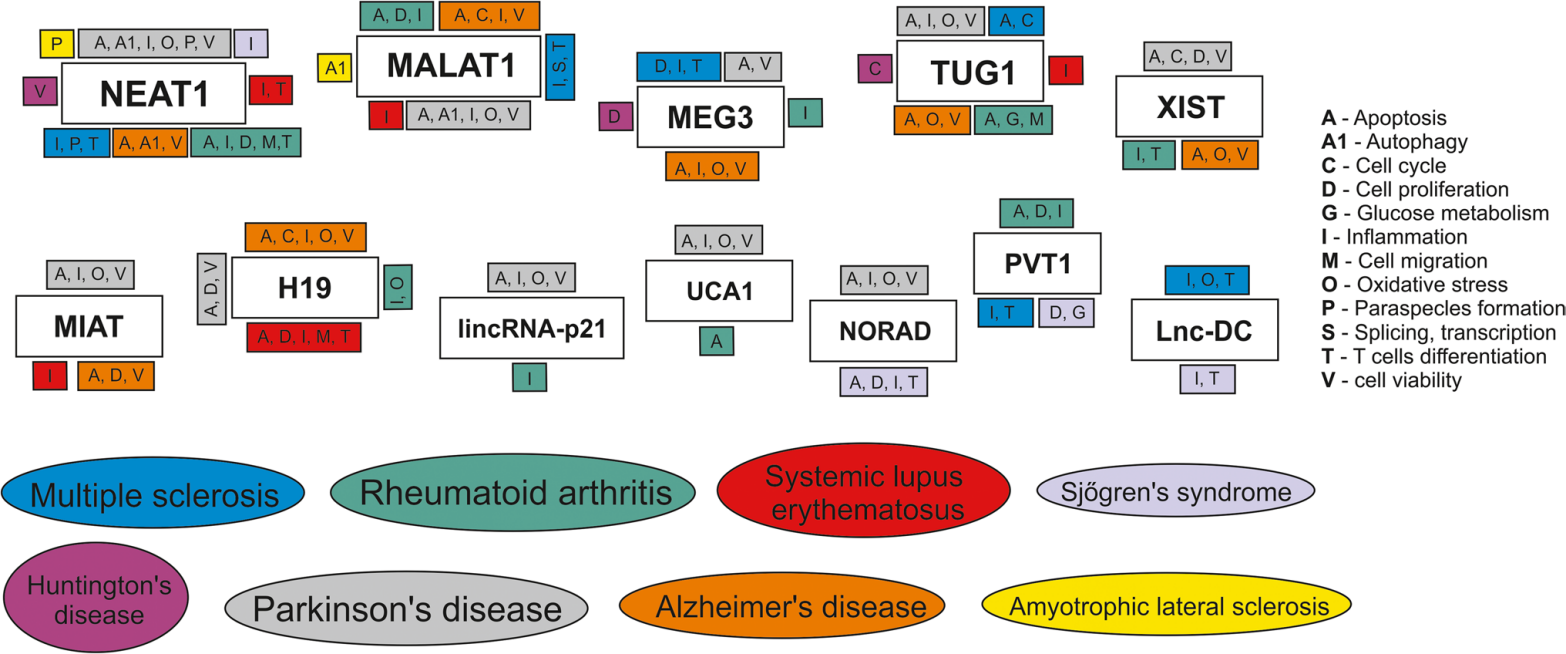
Network of lincRNAs and autoimmune and neurodegenerative diseases. Only lincRNAs involved in more than one disease were considered. Cellular pathways contributing to pathogenesis were shown

Dysregulation of lincRNA expression can abrogate cellular homeostasis, cell differentiation, and development and can also deregulate the immune and nervous systems. Therefore, overexpression, deficiency, or mutation of lincRNA genes has been implicated in genetically inherited disorders and tumorigenic mechanisms, including cell proliferation, migration and invasion, epithelial-to-mesenchymal transition, apoptosis, and cancer metastasis. Considering their involvement in pathological responses, lincRNAs have often been identified to act as miRNA sponges and thereby modulate the expression of both miRNAs and their targets (Fig. 1, Table S1). LincRNAs are strongly related to the progression of various disorders, with examples of individual lincRNAs involved in several different diseases (Fig. 2, Table 1, Table 2). In contrast, the level of a single lincRNA in one disease can vary depending on the type of sample analyzed (Table 1, Table 2). Certain lincRNAs can be considered potential therapeutic targets and valuable biomarkers capable of predicting the onset of a disease, its degree of activity, or the progression phase [2, 3, 5, 6].

**Table 1:** LincRNAs involved in the pathogenesis of autoimmune and neurodegenerative diseases

LincRNA	Regulation	Sample	References
RN7SK RNA	**↑**	Serum (MS)	[9]
LINC00293, RP11-29G8.3	**↑**	Serum (MS)	[10]
Lnc-DC	**↑**	Serum (MS), PBMC (MS), plasma (LN), plasma (SS)	[11, 13, 45, 58]
	↓	Plasma (SLE)	[45]
PANDA	**↑**	PB (MS)	[14]
Linc-MAF-4	**↑**	PBMC (MS)	[16]
AL928742.12	↓	PBMC (MS)	[18]
MEG9	↓	PBMC (MS)	[19]
	**↑**	PBMC (RA), serum (RA)	[26]
HULC	↓	PBMC (MS)	[19]
MIAT (GOMAFU)	↓	PBMC (MS), mouse model (PD, AD), cellular model (PD)	[19, 83, 84, 90]
	**↑**	PB (SLE), cellular model (PD), mouse model (PD)	[54, 85]
PVT1	↓	PB (MS)	[22]
	**↑**	STs (RA), FLSs (RA), CD4^+^ T (SS)	[40, 41, 59]
LincR-Gng2-5′ AS	**↑**	Serum (MS)	[24]
LincR-Epas1-3’AS	↓	Serum (MS)	[24]
Jpx	↓	PBMC (RA)	[26]
lincRNA-p21	↓	PBMC (RA)	[28]
	**↑**	Brains (PD), cellular model (PD), mouse model (PD)	[75–79]
H19	**↑**	STs (RA), marrow-derived mesenchymal stem cells (SLE), PB (PD), cellular model (AD), mouse model (AD)	[29, 53, 82, 92]
	↓	Mouse model (PD), cellular model (PD)	[80, 81]
LERFS	↓	FLSs (RA)	[30]
FER1L4	↓	FLSs (RA), STs (RA)	[31]
GAPLINC	**↑**	FLSs (RA)	[34]
PICSAR	**↑**	FLSs (RA)	[35]
LINC00152	**↑**	FLSs (RA)	[36]
UCA1	↓	FLSs (RA)	[39]
	**↑**	Mouse model (PD), cellular model (PD)	[87, 88]
LINK-A	**↑**	STs (RA), FLSs (RA)	[42]
linc0949	↓	PBMC (SLE)	[44]
linc0597	↓	PBMC (SLE)	[44]
	**↑**	Plasma (SLE)	[45, 46]
lnc0640, lnc5150, lnc6655	**↑**	Plasma (SLE)	[46]
lnc7074	↓	Plasma (SLE)	[46]
RP11-2B6.2	**↑**	Biopsy (SLE)	[47]
TSIX	**↑**	Monocyte-derived dendritic cells (SLE)	[52]
LINC00176	**↑**	CD4^+^ T cells (SLE)	[55]
lincRNA00892	**↑**	CD4^+^ T cells (SLE)	[56]
LINC00511	↓	PBMC (SS)	[60]
NORAD (linc00657)	**↑**	PBMC (SS)	[60]
	↓	Cellular model (PD)	[86]
CYTOR, LINC00426, NRIR	**↑**	PBMC (SS)	[61]
TPTEP1-202	↓	PBMC (SS)	[61]
LINC00487	**↑**	PBMC (SS)	[66]
DGCR5	↓	Brains (HD)	[69]
HAR1	↓	Brains (HD)	[69]
TUNA	↓	Brains (HD)	[71]
LINC00341	**↑**	Brains (HD)	[69]
LINC00342	↓	Brains (HD)	[69]
LINC02470	↓	PB (HD)	[73]
LINC01311	↓	Cellular model (AD)	[91]
LINC00507	**↑**	Cellular model (AD), mouse model (AD)	[93]
LoNA	**↑**	Mouse model (AD)	[94]
E230001N04Rik	**↑**	Cellular model (AD), mouse model (AD)	[95]
CTC-459F4.3, AC003090.1, RP11-219A15.5, LINC01007, MIR7-3HG, LINC00507, LINC00643, LINC01128, CTC-471C19.1, RP11-143M1.3, LINC00672, LINC00460, HAR1F, AC005592.3, CTC-543D15.8, AC007246.3, LINC01123, LINC00461, RP11-45P15.4	↓	Brains (AD)	[96]
LINC00320, RP11-953B20.1, lnc-POTEG-4, lnc-SOX11-3, LINC00844, RP11-274H2.5, NEAT1, LINC01094, XIST	**↑**	Brains (AD)	[96]
CH507-513H4.4, CH507-513H4.6, CH507-513H4.3	**↑**	PBMC (AD)	[97]

*MS*, multiple sclerosis; *RA*, rheumatoid arthritis; *SLE*, systemic lupus erythematosus; *SS*, Sjögren’s syndrome; *HD*, Huntington’s disease; *PD*, Parkinson’s disease; *AD*, Alzheimer’s disease; *ALS*, amyotrophic lateral sclerosis; *PB*, peripheral blood; *PBMC*, peripheral blood mononuclear cells; *LN*, lupus nephritis; *STs*, synovial tissues; *FLSs*, fibroblast-like synoviocytes

**Table 2:** Common lincRNAs implicated in the pathomechanisms of autoimmune and neurodegenerative disorders

Disorder	LincRNA	References
Multiple sclerosis	NEAT1 ↑ (serum, PB)	[9, 14]
	MALAT1 ↑ (PB, serum), ↓ (PBMC)	[11, 19, 126]
	TUG1 ↑ (PB, serum), ↓ (PBMC)	[9, 10, 14, 19]
	MEG3 ↓ (PB, PBMC)	[32, 158]
	XIST ↓ (PBMC)	[19]
Rheumatoid arthritis	NEAT1 ↑ (PBMC, serum, STs)	[26, 102–104]
	MALAT1 ↓ (serum, FLSs)	[26, 128]
	TUG1 ↑ (PBMC, serum, FLSs)	[26, 154]
	MEG3 ↓ (FLSs)	[33]
	XIST ↑ (cartilage tissue)	[163]
Systemic lupus erythematosus	NEAT1 ↑ (PBMC, monocytes, monocyte-derived dendritic cells)	[52, 105, 106]
	MALAT1 ↑ (PBMC, monocytes)	[129]
	TUG1 ↓ (PBMC)	[155]
Sjőgren’s syndrome	NEAT1 ↑ (PBMC)	[109]
Huntington’s disease	NEAT1 ↑ (brains, cellular model, mouse model)	[69, 110–112]
	TUG1 ↑ (brains)	[69]
	MEG3 ↑ (cellular model, mouse model), ↓ (brains)	[69, 110]
	XIST ↑ (cellular model, mouse model)	[110]
Parkinson’s disease	NEAT1 ↑ (PB, brains, cellular model, mouse model)	[113–119]
	MALAT1 ↑ (brains, cellular model, mouse model)	[75, 130–134]
	TUG1 ↑ (serum, cellular model, mouse model)	[150, 151]
	MEG3 ↓ (plasma, cellular model)	[159, 160]
	XIST ↑ (cellular model, mouse model)	[164]
Alzheimer’s disease	NEAT1 ↑ (brains, cellular model, mouse model, rat model)	[120–124]
	MALAT1 ↓ (cerebrospinal fluid and plasma, cellular model, rat model)	[136–138]
	TUG1 ↑ (cellular model, mouse model)	[153]
	MEG3 ↓ (rat model)	[161]
	XIST ↑ (cellular model, mouse model)	[165, 166]
Amyotrophic lateral sclerosis	NEAT1 ↑ (cellular model, mouse model, spinal motor neurons, glial cells)	[143–145, 147]
	MALAT1 (PBMC)	[141]

*PB*, peripheral blood; *PBMC*, peripheral blood mononuclear cells; *STs*, synovial tissues; *FLSs*, fibroblast-like synoviocytes

Knowledge about lincRNAs implicated in human disorders is increasing daily. LincRNAs can play both detrimental and protective pathophysiological roles. This dual nature can be manifested in their ability to directly or indirectly regulate cellular signal transduction pathways and mechanisms that govern basic cellular processes. In this review, we shed light on the implications of lincRNAs in selected autoimmune and neurodegenerative diseases. First, we focus on individual autoimmune and neurodegenerative disorders and exemplify differentially expressed lincRNAs, elucidating their impact on disease pathomechanisms (Table 1). Next, we summarize the activity of lincRNAs that are involved in multiply of these diseases, such as NEAT1, MALAT1, TUG1, MEG3, and XIST (Table 2).

## Autoimmune Disorders

Autoimmune disorders are a group of heterogeneous diseases characterized by inappropriate immune responses to “self” antigens. Consequently, the immune system attacks normal molecules and healthy cells and tissues, causing damage to various organs and systems. LincRNAs may play a crucial role in the pathogenesis of these disorders, as they are important regulators of immune cell differentiation and activation in both the innate and adaptive immune systems. In this chapter, the involvement of lincRNAs in the pathogenesis of multiple sclerosis, systemic lupus erythematosus, rheumatoid arthritis, and Sjögren’s syndrome is described.

### Multiple Sclerosis (MS)

MS is a chronic autoimmune inflammatory neurological disease of the CNS (central nervous system) that leads to sustained disability. The pathogenesis of MS involves genetic susceptibility and environmental factors. The progressive neurodegenerative process includes demyelination, chronic oxidative injury, and eventual axonal neuronal death in the spinal cord, optic nerves, and brain. The adaptive immune system is hyperactivated in the CNS and responsible for the destructive effects of MS. This hyperactivation includes the spontaneous activation of autoreactive proinflammatory T cells, CD4 + Th1 (T helper 1), and Th17 cells, resulting in the secretion of cytokines and stimulation of the inflammatory cascade. In the advanced stages of the disease, the role of B cells in driving inflammation appears to be prominent. Clinically, different MS subtypes have been described: relapsing remitting (RR), secondary progressive (SP), and primary progressive (PP). The pathophysiological mechanisms are associated with genes that play a regulatory role in the immune system [8].

To search for lincRNAs as biomarkers, in 2016, Santoro et al. screened 84 lncRNAs involved in autoimmunity and the human inflammatory response in serum from 12 patients with RRMS and 12 healthy controls and found lincRNAs that were upregulated in patients with RRMS and could play a role in neurodegenerative processes of MS, among others **RN7SK RNA** (the 7SK small nuclear RNA). RN7SK RNA is a part of the 7SK snRNP complex and represses the cellular kinase complex P-TEFb (positive transcription elongation factor b), which regulates the activity of CD4 + T lymphocytes. Upregulation of RN7SK RNA leads to disruption of CD4 + T cell differentiation and contributes to inflammation and activation of the autoimmune process [9]. Similarly, upregulation of **LINC00293** and **RP11-29G8.3** was reported in serum obtained from 16 SPMS and 12 PPMS Italian patients compared to 8 healthy controls [10]. Next, by in silico prediction, the authors identified the possible interactions of these lincRNAs with miRNAs and their putative targets possibly involved in MS. Among these interactions, miR-200a, miR-141, miR-24-3p, miR-15a, and miR-15b can be sequestered by RP11-29G8.3 (Table S1). Consequently, the levels of these miRNAs were decreased in blood samples from MS patients, causing the induction of their targets involved in the inflammatory response [10].

The lincRNA **lnc-DC** was also upregulated in serum obtained from 45 RRMS patients compared to 45 controls [11]. The lincRNA lnc‑DC is exclusively expressed in human DCs (dendritic cells) and is involved in their differentiation and maturation. In the cytoplasm, lnc‑DC directly binds to STAT3 (signal transducer and activator of transcription 3) and promotes its phosphorylation on tyrosine 705 [12]. Transcription factor STAT3 regulates many immune-associated genes and plays a role in Th17 cell differentiation. Lnc-DC overexpression could induce the overmaturation of dendritic cells via STAT3. These activated cells further migrate across the blood–brain barrier and stimulate the differentiation of memory T cells into proinflammatory Th1 and Th17 lymphocytes. In addition, macrophages and microglial cells are activated and produce other proinflammatory cytokines, oxygen radicals, and nitric oxide radicals responsible for demyelination and axonal loss [12]. Recently, a higher lnc-DC expression was confirmed in the PBMCs (peripheral blood mononuclear cells) of 50 female patients who were negative for the HLA-DRB1*15:01 allele, suggesting that lnc-DC expression is sex-specific among multiple sclerosis patients [13]. LincRNA expression regulation in MS by sex-determined factors and hormones was further suggested in case of **PANDA** (P21-associated ncRNA DNA damage activated). Overexpression of PANDA was observed in PB (peripheral blood) samples from 50 patients with RRMS compared to 50 healthy controls, with more prominent overexpression in male patients [14]. PANDA has an antiapoptotic effect and stabilizes the p53 protein after DNA damage induction [15].

By microarray analysis of PBMCs from 6 patients with RRMS compared to 5 healthy controls, scientists identified increased expression of **linc**-**MAF-4** and correlated its level with the annual relapse rate in MS patients [16]. Linc-MAF-4 negatively regulates the transcription of MAF (V-maf musculoaponeurotic fibrosarcoma oncogene homolog), a Th2-associated transcription factor, by recruiting chromatin modifiers to sites within the genomic region of *MAF*. Downregulation of MAF leads to inhibition of T cell differentiation into Th2 cells. Linc-MAF-4 also promotes the activation of CD4 + helper T lymphocytes from patients with MS [16, 17].

Instead, by comparing PBMCs from 50 patients with RRMS with those from 25 healthy controls, Ghoveud et al. found downregulation of a memory B cell lineage-specific lincRNA, **AL928742.12**. Its level correlated with the depletion of its *cis*-regulated target, IGHA2 (immunoglobulin heavy constant alpha 2). *IGHA2 *encodes the constant region of heavy immunoglobulin chains and is involved in the differentiation of B lymphocytes during the humoral immune response [18]. Similarly, downregulation of the lincRNAs **MEG9** (maternally expressed 9), **HULC** (hepatocellular carcinoma upregulated long noncoding RNA), and **MIAT** (myocardial infarction associated transcript, also called GOMAFU) was reported by Fenoglio et al. who studied PBMC samples from 27 patients with RRMS, 13 patients with PPMS, and 31 healthy controls in the Italian population [19]. MIAT can bind to the splicing factor SF1 (splicing factor 1), inhibiting splicing by preventing the formation of spliceosome complexes [20]. MIAT is expressed predominantly in the CNS, where it regulates the differentiation of neural stem cells into oligodendrocytes [21].

Another study carried out in circulating blood cells from 50 Iranian patients with RRMS, and 50 healthy controls showed downregulation of **PVT1** (plasmacytoma variant translocation 1) with a moderate correlation between the level of expression of PVT1 and the duration of the disorder and no significant correlation with either the EDSS (expanded disability status scale) score or age at onset [22]. There are some features that put PVT1 in an interaction network with a major role in the pathogenesis of MS: (i) The *PVT1* gene is located in a genomic region (8q24) that is a preferred site for chromosomal rearrangements in cancers and contains multiple risk loci for different diseases, including MS; (ii) PVT1 controls the release of IL-6 (interleukin 6); (iii) PVT1 functions as a molecular sponge for mir-200 family members, including miR-200a, which mediates the pathogenesis of MS through promoting the differentiation of Th17 cells and preventing the differentiation of Treg (regulatory T cells) cells (Table S1); and (iv) the main target of PVT1 is the MYC transcription factor, which is required for Th17 differentiation [22].

Two lincRNAs, **LincR-Gng2-5’AS** (transcribed in Th1 cells by the transcription factor STAT4) and **LincR-Epas1-3’AS** (transcribed in Th2 cells by STAT6), are located in genomic regions rich in genes encoding proteins that regulate the expression of cytokines and various immune factors [23]. A study conducted on serum from 42 RRMS and 18 SPMS Egyptian patients compared to 60 healthy controls showed upregulation of LincR-Gng2-5′AS in Th1 cells and downregulation of LincR-Epas1-3′AS in Th2 cells. This deregulation was more marked in patients with SPMS, and these two lincRNAs showed opposite correlations with the EDSS score (positive for LincR-Gng2-5′AS and negative for LincR-Epas1-3′AS) [24]. Changes in the expression of these two lincRNAs may play a role in the pathogenesis of MS for two reasons: (i) Both lincRNAs can regulate the expression of nearby genes that encode various immune regulators, and (ii) increased proinflammatory activity of Th1 cells and decreased anti-inflammatory activity of Th2 cells, which is correlated with the levels of both lincRNAs, have been observed in multiple sclerosis [23, 24].

### Rheumatoid Arthritis (RA)

RA is characterized by chronic inflammation, and destruction of synovial joints and is caused primarily by the release of inflammatory cytokines, including TNF-α (tumor necrosis factor α), IL-1β, and IL-6. The major cellular contributors in RA are T and B lymphocytes, neutrophils, macrophages, and proliferating FLSs (fibroblast-like synoviocytes). The pathological changes include immune cell-mediated cartilage and subchondral bone damage, which can lead to joint deformities. FLSs play a critical role in the initiation of RA. Their abnormal activation, apoptosis resistance, and tumor-like transformation have been regarded as key steps in joint destruction [25].

Microarray analysis of lncRNAs in PBMCs and serum exosomes from 28 RA patients compared to healthy controls revealed lincRNA **MEG9** that was significantly upregulated in both kinds of RA samples, whereas a decreased level of the lincRNA **Jpx** was observed in PBMC samples [26]. The lincRNA Jpx is known as a molecular switch for X-chromosome inactivation but also functions as an oncogene in cancer cells, inducing cell proliferation and cell migration [27]. In another study, Spurlock et al. detected a decreased level of **lincRNA-p21** and an increased level of phosphorylated p65, a marker of activation of the proinflammatory transcription factor NF-κB (nuclear factor-kappa B), in the PBMC samples from 8 patients with RA compared to 9 healthy individuals. Interestingly, lincRNA‑p21 expression was induced in patients with RA treated with methotrexate, and this effect was further confirmed in cultured cells. LincRNA‑p21 binds to its target mRNAs and represses their translation by recruiting the translational repressor DDX6 (DEAD box protein 6). It potentially also represses proteins critical for NF‑κB transcriptional activity; therefore, lincRNA-p21 reduction may contribute to inflammation [28].

Analysis of synovial specimens (macrophages and fibroblasts) isolated from 26 patients with RA, 25 patients with osteoarthritis, and 2 patients with reactive arthritis compared with 15 control samples showed significant overexpression of the lincRNA **H19** in the RA samples and osteoarthritis samples [29]. H19 RNA is abundantly expressed in endodermal and mesodermal-derived embryonic tissue, and after birth, its expression is repressed in all tissues except skeletal muscle. However, reexpression of H19 was observed in several different tumors; therefore, H19 RNA has been proposed as a marker of embryonal dedifferentiation of adult tissues and as a tumor marker. H19 upregulation mimics inflammatory/oxidative stress. Among the H19-regulated targets, genes encoding pathological stress factors, such as thioredoxin, MKK1 (MAP-kinase kinase 1), NF-κB, JNK2, TNF-α, and IL-6, have been identified, and these factors may contribute to inflammation in RA. Therefore, H19 upregulation mimics inflammatory and oxidative stress and/or dedifferentiation of adult synovial tissues, which could underlie the pathogenesis of RA [29].

Microarray analysis of 3 samples of FLSs from both healthy controls and patients with RA revealed significant downregulation of the lincRNA **LERFS** (lowly expressed in RA FLSs) in RA FLSs (FLSs isolated from RA patients). In vitro experiments in cultured FLSs and further in vivo experiments using mouse models showed that LERFS negatively regulates RA FLS migration, invasion, and proliferation through interaction with hnRNPQ (heterogeneous nuclear ribonucleoprotein Q) and that this LERFS-hnRNPQ complex controls mRNA metabolism and cell motility by modulating the stability or translation of the *RhoA*, *Rac1,* and *CDC42* mRNAs [30]. Another lincRNA that might regulate inflammation in RA FLSs is **FER1L4** (Fer-1-like protein 4). Yu et al. examined the expression of FER1L4 and NLRC5 (nucleotide oligomerization domain-like receptors 5) and the mRNA and protein levels of inflammatory cytokines in FLSs and STs (synovial tissues) from patients with RA. They observed that the *FER1L4* gene promoter was significantly methylated in RA FLSs, which led to a reduction in the FER1L4 level [31]. This in turn corresponded to an increased level of NLRC5 [32]. NLRC5 is a key regulator of the adaptive immune response stimulating the production of inflammatory cytokines, and its deregulation has also been linked to the pathogenesis of rheumatoid arthritis [31, 33].

In contrast, the levels of the lincRNAs **GAPLINC** [34] and **PICSAR** [35] are increased in RA FLSs, as verified by analyses of 11 and 8 patients with RA, respectively, compared with patients with common traumatic injury. Both factors act by sponging miRNAs, namely, miR-382-5p, miR-575, and miR-4701-5p (Table S1), and their upregulation promotes cell proliferation, migration, and invasion and induces the production of proinflammatory cytokines and proteinases, including IL-6, IL-8, and MMP-9 (matrix metalloproteinase-9), in RA-FLSs [34, 35]. In another study, lncRNA microarray analysis was applied to identify differentially expressed lncRNAs in RA FLSs. The cytoskeleton regulator **LINC00152** was shown to be upregulated in RA FLSs, inducing the proliferation but suppressing the apoptosis of these cells. Furthermore, research showed a positive feedback loop between LINC00152 and FOXM1, which promoted the growth of RA FLSs via the Wnt/β-catenin pathway [36]. This signaling cascade is involved in cell proliferation, differentiation, survival, migration, motility, and polarity. In addition, it is known to modulate immune responses during inflammation. Therefore, aberrant Wnt signaling mediates the development and progression of cancer, inflammatory and immune diseases, and metabolic and neurodegenerative disorders [37, 38]. The Wnt signaling pathway is also targeted by the lincRNA **UCA1** (urothelial cancer associated 1). In RA FLSs, the level of UCA1 was decreased, which increased RA FLS viability via decreased expression of Caspase-3 and inhibition of apoptosis. Moreover, scientists have found an inverse correlation between the levels of UCA1 and Wnt6, which induces the Wnt signaling pathway in RA FLSs and thus promotes RA progression [39].

Zhang et al. established rat models of RA to determine the mechanisms of inflammation and apoptosis in synovial tissues and FLSs. They reported high expression of the lincRNA **PVT1** and low expression of its target, SIRT6 (Sirtuin 6), in both tissues and FLSs of RA. PVT1 binds to the *SIRT6* promoter to induce its methylation, which in turn inhibits *SIRT6* transcription. SIRTs are regulators of many processes, including cell survival, gene transcription, and inflammation. Therefore, decreased SIRT6 activity in RA FLSs is thought to induce cell proliferation and inflammation and suppress apoptosis [40]. Recently, increased PVT1 expression was confirmed in STs from 30 patients with RA. Concurrently, the level of its target, miR-145-5p, was significantly decreased, resulting in enhanced cell proliferation and increased secretion of IL‐1β and IL‐6 in RA cells (Table S1) [41]. Higher expression of **LINK-A** was also recently observed in STs and FLSs from 5 patients with RA compared with 5 healthy controls and was positively correlated with the severity of synovitis in the patients. LINK-A was shown to regulate inflammation and invasion through the HIF-1α signaling pathway, inducing the expression and secretion of matrix metalloproteinases and proinflammatory cytokines, such as IL-1β, IL-6, and IL-8. LINK-A knockdown decreased the migration and invasion of RA FLSs [42].

### Systemic Lupus Erythematosus (SLE)

SLE is a chronic systemic autoimmune disease with a high incidence in women of childbearing age. SLE is characterized by loss of immunological tolerance to self-nuclear antigens, production of multiple autoantibodies, excessive proinflammatory cytokine and chemokine production, and, ultimately, damage to tissues or organs, most commonly the skin, kidney, lung, nervous system, and circulatory system. Disruption of monocytes initiates the autoreactive cascade in SLE. Moreover, T cells, B cells, and dendritic cells are crucial cells for SLE pathogenesis. LN (lupus nephritis) is one of the most serious complications of SLE and one of the leading causes of morbidity and mortality in patients [43].

Wu et al. showed that the expression of **linc0949** and **linc0597** was significantly decreased in PBMCs from 102 patients with SLE compared to 76 healthy donors [44]. Further analysis associated linc0949 with the incidence of LN and revealed a correlation between the linc0949 level and disease activity, as assessed by both the SLEDAI (SLE disease activity index) score and the level of complement component C3, in patients with SLE. Furthermore, linc0949 expression was found to be decreased in patients with SLE with ongoing or cumulative organ damage. In three patients with severe disease flares, linc0949 expression increased significantly after treatment with immunosuppressive agents, suggesting its responsiveness to therapy and making it a good candidate as a biomarker for diagnosing and for guiding SLE therapy [44]. In another study conducted in two independent cohorts, plasma from healthy controls and SLE patients divided into those with lupus nephritis and those with SLE without nephritis was analyzed. The results revealed that the level of **linc0597** was increased in all patients with SLE [45], in contrast to observations in PBMCs [44]. Furthermore, the level of **lnc-DC** was significantly higher in the patients with LN compared to patients with SLE without nephritis but was significantly decreased in all SLE patients compared with healthy controls. Therefore, linc0597 has been suggested as a biomarker to identify patients with SLE, while lnc-DC could be used to discriminate LN from SLE without nephritis [45]. Wu et al. examined plasma samples from patients with SLE and healthy controls as well [46]. The levels of **linc0597**, **lnc0640**, and **lnc5150** were elevated in SLE patients, whereas that of **lnc7074** was decreased. Furthermore, they showed that the expression levels of lnc0640, lnc5150, **lnc6655**, and lnc7074 were significantly higher in patients with LN than in those without LN, as previously shown for linc0597 and lnc-DC [45]. Therefore, the plasma levels of these six lincRNAs may reflect the renal pathology of SLE. Moreover, comparison of lincRNA expression levels in plasma from 30 patients with rheumatoid arthritis and 31 patients with primary Sjögren’s syndrome with plasma from SLE patients indicated that the combination of lnc7074, linc0597, lnc0640, and lnc5150 could distinguish SLE from the other two diseases. KEGG pathway analysis revealed that lnc0640 and lnc5150 may participate in the development of SLE through the MAPK (mitogen-activated protein kinase) signaling pathway, which regulates the immune response of T and B cells as well as the production of multiple SLE-related inflammatory factors, such as TNF-α, IL-1/6, and IFN. In addition, lnc0640, lnc6655, and lnc7074 could regulate the expression of their predicted target genes by acting as ceRNAs (competing endogenous RNAs) for the target miRNAs [46].

RNA-seq of renal biopsy samples from 22 patients with LN and 7 controls revealed an elevated level of the lincRNA **RP11-2B6.2**, with higher expression in LN patients with active lesions than in those with chronic lesions. RP11-2B6.2 has been shown to function as a positive regulator of the IFN-I (type I interferon) signaling pathway through epigenetic inhibition of its negative regulator, the *SOCS1* (suppressor of cytokine signaling 1) gene. RP11-2B6.2 overactivates the IFN-I pathway, which in turn results in activated phosphorylation of JAK1 (Janus kinase 1), TYK2 (tyrosine kinase 2), and STAT1 [47]. The IFN-I signaling pathway is a crucial component of the innate and adaptive immune responses. Interferons exhibit antiviral activity as well as growth inhibitory and immunomodulatory effects by modulating the expression of ISGs (IFN-stimulated genes) [48, 49]. Type I IFNs are considered to play a pathogenic role in autoimmune diseases [50].

Recently, global comprehensive analyses using microarrays or high-throughput RNA sequencing have been performed, providing a new direction for the diagnosis and treatment of SLE by suggesting lincRNAs that might serve as biomarkers. In 2019, Ye et al. performed RNA-seq of PBMCs from 147 individuals with SLE and 117 healthy donors and identified 23 lincRNAs that were differentially expressed and may be related to SLE. They constructed a coexpression network between the lincRNAs and their predicted target genes, which further allowed verification of the possible biological functions of these 23 lincRNAs in metabolism, cellular signal transduction, the cell cycle, apoptosis, cell adhesion, or antigen presentation [51]. In another study, moDCs (monocyte-derived dendritic cells) from 15 female SLE patients were compared with those from 15 female healthy controls. A total of 150 differentially expressed lincRNAs were identified, for example, the highly upregulated lincRNA **TSIX** (XIST antisense transcript). Its expression was positively correlated with the SLEDAI score. Upregulated TSIX facilitates X chromosome inactivation by protecting the active X from ectopic silencing, consistent with the increased incidence and prevalence of SLE in women [52].

Among the accumulating recent reports, the implication of the lincRNAs **H19** and **MIAT** in SLE was discovered, which both have been already connected to immunological disorders, RA and MS, respectively [19, 29]. In SLE, upregulation of H19 and MIAT induces dysregulation of the immune system by inhibiting IL-2 production and of the complement system by sponging miRNAs, respectively (Table S1) [53, 54]. In turn, overexpressed **LINC00176** and **lincRNA00892** activate CD4 + T cells and promote their adhesion and proliferation, respectively, in SLE patients [55, 56].

### Sjögren’s Syndrome (SS)

SS is an autoimmune disease characterized by inflammation and reduced secretory function of the exocrine glands, mainly the salivary and lachrymal glands, leading to dry mouth and dry eye symptoms. This inflammation is caused by the infiltration of lymphocytes and plasma cells, mainly CD4 + T cells, which can form inflammatory foci in the salivary and lacrimal glands of SS patients. High proliferation rates and hyperactivation of CD4 + T and B cells lead to hypergammaglobulinemia, circulating immune complexes, and the production of autoantibodies against SS-related antigens A and B (anti-SSA and anti-SSB, respectively). SS affects ~ 1% of the general population, mainly females, with relatively high morbidity among autoimmune diseases. SS is subclassified as pSS (primary SS) when it develops in isolation or as sSS (secondary SS) when it develops in combination with another systemic autoimmune rheumatic disorder, such as SLE or RA [57].

Chen et al. analyzed the **lnc-DC** level in the plasma of 127 pSS patients without ITP (immune thrombocytopenia), 22 pSS patients with ITP, 50 patients with SLE, 50 patients with RA, and 109 healthy individuals and reported that the lnc-DC level was significantly elevated in pSS patients, especially in pSS patients with ITP. The lnc-DC expression level was positively correlated with some clinical pSS characteristics, including the β_2_-microglobulin, anti-SSA and anti-SSB antibody levels, and the erythrocyte sedimentation rate [58]. The researchers suggested that through STAT3 regulation, lnc-DCs can induce Th17 cell differentiation and, therefore, the secretion of a variety of cytokines is involved in the occurrence and development of pSS [12, 58]. Moreover, scientists found that the combined analysis of lnc-DC and anti-SSA/SSB antibody levels significantly improved the diagnostic ability and could be used to distinguish pSS patients from SLE and RA patients [58].

Another study focused on the lincRNA **PVT1**, which was selected after microarray analysis of RNA isolated from samples of labial glands from 30 SS patients and 16 controls. Deregulation of PVT1 expression has already been reported in MS and RA [22, 40, 41]. In patients with SS, PVT1 was upregulated in CD4 + T cells, and its expression was also induced in healthy donor CD4 + T cells activated by antigen simulation. Furthermore, it was demonstrated that under such antigen stimulation, PVT1 induces the expression of the transcription factor MYC, which, upon T cell activation, in turn controls metabolic reprogramming toward enhanced glucose and amino acid metabolism, as required by proliferating activated T cells [59]. Moreover, Dolcino et al. analyzed the expression profile of ~ 50,000 lncRNAs in a sample of PBMCs derived from 8 female patients with SS and 8 healthy controls using microarrays as well. After complex network analysis, they identified some functional interactions of lincRNA-miRNA pairs with target genes involved in biological processes and molecular pathways crucial to the pathogenesis of pSS. Among the lincRNAs, **LINC00511** (downregulated) and **NORAD** (noncoding RNA activated by DNA damage) (upregulated) were related to the most transcripts related to the immune response, T and B cell development and function, malignancy, inflammation, apoptosis, INF-I signaling, and epithelial cell adhesion and polarization, as well as signaling pathways involved in salivary gland morphogenesis [60]. In turn, RNA-seq was applied to identify lncRNAs and mRNAs that were differentially expressed in PBMCs from 5 pSS patients compared to 5 healthy controls and showed that **CYTOR** (cytoskeleton regulator RNA), **LINC00426**, and **NRIR** (negative regulator of IFN response) were significantly upregulated, but **TPTEP1-202** was significantly downregulated in pSS patients. Their expression was strongly related to the ESSDAI (EULAR Sjögren’s syndrome disease activity index) score and the serum IgG, CRP (C-reactive protein), and C4 levels, which were closely correlated with the disease activity of pSS. Coexpression and colocalization analyses revealed that differentially expressed lincRNAs in the PBMCs of patients with pSS were strongly correlated with deregulation of mRNAs functioning in pathways related to the development and differentiation of immune cells, to the regulation of inflammatory cytokine release and to cell metastasis and signaling, including the NF-κB, MAPK, and JAK-STAT pathways [61], which are crucial mediators of immune and inflammatory responses [62–65].

Recently, by studying PBMC samples from 6 patients with SS and 6 controls, Inamo et al. identified that **LINC00487** was upregulated in subsets of B cells from patients with SS and found that its level was correlated with the disease score and the expression levels of genes in the IFN signaling pathway. The expression of LINC00487 in B cells is induced by IFNα, which promotes the autoreactivity of B cells through germinal center pathways. Furthermore, since LINC00487 was found to be upregulated in the subgroup of germinal center B cells, scientists suggested that LINC00487 expression reflects or even regulates the enhancement of a germinal center-like reaction by IFNα beginning at an early stage of B cell development, which consequently leads to B cell autoreactivity in patients with pSS [66].

## Neurodegenerative Diseases

Neurodegenerative diseases are a group of biologically heterogeneous and complex disorders characterized by the progressive death of neuronal cells in the nervous system that are crucial for mobility, coordination, speech, memory, sensation, and cognition. Both genetic and environmental risk factors contribute to the etiology of these symptoms. Moreover, they exhibit common histopathological lesions of misfolding and aggregation of specific proteins inside or outside cells. Currently, there are no effective treatments for neurodegenerative diseases. However, extensive studies incorporating patient-derived iPSCs (induced pluripotent stem cells) and cellular and animal models that reproduce at least some disease-relevant phenotypes are being conducted to identify diagnostic biomarkers and molecular targets for therapies [67]. Here, we summarize the emerging functions of lincRNAs in RNA networks and signaling pathways—including apoptosis, mitochondrial dysfunction, and inflammatory reactions—that are associated with neurodegeneration in Huntington’s disease, Parkinson’s disease and Alzheimer’s disease.

### Huntington’s Disease (HD)

HD is an adult-onset, autosomal dominant hereditary neurodegenerative disease characterized by cognitive deficits, involuntary movements, and neuropsychiatric changes. It is caused by expansion of a CAG repeat (> 36 repeats) encoding a polyglutamine (polyQ) tract in the first exon of the Huntingtin (*HTT*) gene. The polyQ tract is responsible for the toxic gain of function and aggregation of mutant Huntingtin (mHTT) protein, leading to neuronal dysfunction and loss. The major and earliest site of pathology is the striatum, with medium spiny neurons the most vulnerable cells in HD; however, other regions of the brain, such as the cortex and cerebellum, are also affected [68].

In HD, lincRNAs have been associated with different aspects of disease-relevant mechanisms. One such mechanism involves the neuronal-specific transcriptional silencer REST (repressor element 1-silencing transcription factor), which represses a wide array of neural-specific genes that play essential roles in nervous system development and function. In healthy individuals, REST is sequestered in the cytoplasm by wild-type Huntingtin (wtHTT). This interaction is weakened in the case of mHTT, leading to increased trafficking of REST to the nucleus, where it can exhibit its repressive activity through binding to NRSEs (neuron-restrictive silencer elements) in the promoters of target neuronal genes and recruitment of other components of the repressor complex. These events result in chromatin structural changes and inhibited gene transcription [67]. *BDNF* (brain-derived neurotropic factor) gene is one of the best-characterized protein-coding targets of REST, but there are also lincRNAs that are known to be directly regulated by REST and affected in HD brains: DGCR5 (DiGeorge syndrome critical region gene 5) and HAR1 (human accelerated region 1). **DGCR5** is a neural-specific lincRNA with decreased expression in the caudate nucleus of HD brains [69]. Its overexpression and negative effect on proapoptotic PRDM5 (PR/SET Domain 5) was shown to inhibit neuronal apoptosis and alleviate acute spinal cord injury in a rat model [70], suggesting a potential neuroprotective role of DGCR5 in the neurodegeneration in HD. In turn, human accelerated region 1 is a conserved genomic region transcribed into a *cis*-antisense pair of structured lincRNAs, **HAR1F and HAR1R**, which are specifically expressed in developing cortical neurons [68]. Global ChIP-seq analysis identified HAR1 as a REST target and, consistent with this finding, showed that both HAR1F and HAR1R are significantly downregulated in the striatum of HD patients [69]. However, the role of these lincRNAs in neurodegenerative diseases is still obscure.

**TUNA** (Tcl1 upstream neuron-associated lncRNA) exhibits CNS-specific expression in vertebrates. This lincRNA forms a complex with RNA-binding proteins that is enriched at the promoters of the *Sox2*, *Nanog*, and *Fgf4* genes. Functional studies performed on mouse embryonic stem cells revealed its essential roles in pluripotency and neural differentiation. Global transcriptome analysis of genes affected by TUNA knockdown showed extensive dysregulation of genes associated with neurodegenerative diseases. Via microarray gene expression profiling of 4 brain regions in 44 HD patients and 36 healthy individuals, the authors demonstrated that TUNA expression is decreased in HD cells and inversely correlated with pathological disease severity. Interestingly, this effect was observed only in the striatum (exhibiting high expression of TUNA and Sox2 in healthy brains), whereas TUNA expression in the motor cortex, prefrontal association cortex, and cerebellum was comparable between HD patients and controls [71].

High-throughput transcriptome profiling of 20 postmortem human HD brains and 49 control brains revealed that 6.8% of the differentially expressed genes were lincRNAs [72]. Reanalysis of microarray data from HD and healthy brains identified two lincRNAs with deregulated expression in HD with a currently unknown role in the etiology of the disease. **LINC00341**, expressed mainly in adipose tissue, was upregulated, while brain-specific **LINC00342** showed downregulation in HD caudates [69]. In addition, using an RNA-seq approach, Colpo et al. identified **LINC02470** to be downregulated in peripheral blood samples collected from 16 patients with a genetic diagnosis of HD in comparison to 8 healthy individuals [73].

### Parkinson’s Disease (PD)

PD is a progressive neurodegenerative disorder that affects approximately 2–3% of elderly people. The underlying pathology involves the selective loss of dopaminergic neurons in the SN (substantia nigra) of the brain and the intracellular aggregation of α-synuclein (α-syn) in Lewy bodies, leading to motor dysfunction and cognitive decline. The vast majority of PD cases are sporadic, with both genetic and environmental factors playing a role. Only approximately 10% are familial, arising through mutations in mitochondria-associated genes, including *SNCA* (α-synuclein), *LRRK2* (leucine-rich repeat kinase 2), *Parkin* (PARK2), *PINK1* (phosphatase and tensin homolog induced putative kinase 1), *PARK7* (protein deglycase DJ-1), and *ATP13A2* (ATPase type 13A), pointing to the important role of mitochondrial dysfunction in the pathogenesis of PD [74]. LincRNAs are involved in different pathological pathways and mechanisms of PD, such as α-syn proteostasis, mitochondrial dysfunction, oxidative stress, apoptosis, and neuroinflammation.

Expression profiling of 20 PD brains and 10 control brains revealed the essential upregulation of **lincRNA-p21** in PD [75]. Furthermore, Ding et al. reported an elevated level of lincRNA-p21 in SH-SY5Y cells treated with MPP^+^ (1-methyl-4-phenylpyridinium) to establish an in vitro model of PD. The high level of lincRNA-p21 resulted in decreased cell viability and increased cytotoxicity, apoptosis, oxidative stress, and neuroinflammation. Further research revealed a new lncRNA-miRNA-mRNA regulatory network in PD with lincRNA-p21 acting as a miR-625 sponge and thus indirectly derepressing the gene encoding the Ca^2+^ channel TRPM2 (Table S1). Activation of TRPM2 leads to cytotoxic influx of Ca^2+^ and, in turn, neuronal death [76]. Additionally, by functioning as a miR-1277-5p sponge, lincRNA-p21 positively regulates the expression of the miR-1277-5p target α-syn (Table S1) [77]. Another study revealed the role of lincRNA-p21 in sustained neuroinflammation mediated by excessive activation of microglia, which is one of the essential initiating factors in the onset and progression of PD. LincRNA-p21 promotes LPS (lipopolysaccharide)-induced microglial activation via a p53-dependent pathway by regulating the miR-181 family/PKC-δ (protein kinase C-delta) axis (Table S1) [78]. Recently, it was shown that upregulation of lincRNA-p21 regulates the degradation of TGIF1 (TG-interacting factor 1) through the (STAU1)-mediated mRNA decay (SMD) pathway in a cellular model of PD. TGIF1 was identified as a transcriptional regulator of α-syn in PD [79].

In turn, the lincRNA **H19** has been reported to be downregulated in PD mice and in cellular models [80, 81]. H19 can rescue dopaminergic neuron loss by sponging miR-301b-3p and thus indirectly increasing the level of HPRT1 (hypoxanthine–guanine phosphoribosyltransferase) (Table S1). HPRT1 mediates the activation of the Wnt/β-catenin signaling pathway, which plays essential roles in dopaminergic neuron development [81]. Moreover, H19 can inhibit apoptosis via modulation of the miR-585-3p/PIK3R3 (phosphoinositide-3-kinase regulatory subunit 3) axis (Table S1) [80]. In contrast to the above findings, H19 expression was found to be upregulated in PB samples of PD patients [82]. Next, **MIAT** downregulation has been reported in different brain regions in PD mice as well as in cellular models [83, 84]. Further study demonstrated that MIAT can regulate SYT1 (synaptotagmin-1) expression by sponging miR-34-5p (Table S1). SYT1 regulates neuronal exocytosis, and its downregulation has been suggested to play a role in dopaminergic neuron degeneration in PD. Moreover, MIAT exerts its neuroprotective effect by promoting the expression of Parkin and tyrosine hydroxylase and decreasing apoptosis [84]. Xu et al. found that MIAT can suppress neuronal damage by modulating the miR-132/SIRT1 axis (Table S1) [83]. In contrast, increased expression of MIAT was reported in a mouse model of PD and in MN9D cells stimulated with MPP^+^. The authors showed that by acting as a miR-221-3p sponge, MIAT can derepress its target TGFBR1 (TGFB receptor 1) and deregulate TGF-β1 (transforming growth factor) and Nrf2 (nuclear factor E2-related factor 2) expression to promote apoptosis, neuroinflammation, and oxidative stress in PD (Table S1). TGF-β1 is involved in microglial activation, which plays an important role in PD, and the activation of the Nrf2 pathway has been proposed to mitigate the neurodegenerative process in PD [85].

Another lincRNA, **NORAD**, has been found to be downregulated in MPP^+^-induced neuroblastoma cell models of PD. Mechanistic studies revealed that NORAD protects cells against MPP^+^-induced destruction, oxidative stress, and inflammatory responses by acting as a miR-204-5p sponge and thus indirectly regulating the level of its target SLC5A3 (solute carrier family 5 member 3) (Table S1), which is downregulated in the putamen in PD [86]. In turn, abnormal **UCA1** expression has been demonstrated in a mouse model of PD and further suggested to facilitate PD progression by influencing the α-syn abundance [87]. Recently, the role of UCA1 in MPP^+^-induced cytotoxicity has been linked to the miR-423-5p/KCTD20 (potassium channel tetramerization domain containing 20) axis (Table S1). Both UCA1 and KCTD20 were found to be upregulated in PD patients [88].

### Alzheimer’s Disease (AD)

AD is a progressive, uncurable neurodegenerative disease particularly affecting people over 65 years old and clinically manifesting as the loss of intellectual and social skills. The reduction in brain volume observed in AD patients is mainly due to hippocampal degeneration, and the main pathological hallmarks of AD include amyloid-beta (Aβ) deposits that form plaques in the extracellular space and neurofibrillary tangles formed by hyperphosphorylated Tau protein (pTau). Amyloid-beta peptide is produced through sequential cleavage of a transmembrane protein, APP (amyloid precursor protein), by BACE1 (β-site APP cleaving enzyme) and γ-secretases. Excessive accumulation of Aβ in the brain has been proposed to be an early neurotoxic event in the pathogenesis of AD [89]. Accumulating evidence shows that lincRNAs exhibit expression abnormalities during AD progression and are involved in the major mechanisms that underlie AD pathology, including regulation of Tau, production and accumulation of Aβ peptide, inflammation, and cell death.

**MIAT** expression was significantly downregulated in the brain parenchyma of Alzheimer’s disease transgenic mice. MIAT knockdown via MIAT shRNA injection into the hippocampus of AD mice resulted in deterioration of memory and learning abilities. Physiologically, these effects were accompanied by increases in the Aβ_40_ and Aβ_42_ levels and neuronal death as well as a decrease in the number of brain microvessels. The authors also showed that MIAT is involved in amyloid clearance due to its effect on LRP1 (low-density lipoprotein receptor related protein 1), which is an important Aβ clearance transporter at the blood–brain barrier [90]. Moreover, reduced expression of **LINC01311** was recently detected in a cellular model of AD. Further experiments showed that LINC01311 promotes proliferation and reduces apoptosis and autophagy by sponging miR-146a-5p (Table S1). Consistent with these findings, upregulation of this miRNA was observed in AD patients and AD animals [91].

In turn, in mouse and cellular models of AD, Zhang et al. observed an elevated level of lincRNA **H19** along with downregulated expression of miR-129 and upregulated expression of the miR-129 target, mRNA encoding the proinflammatory cytokine HMGB1 (high-mobility group box 1) (Table S1) [92]. HMGB1 activation was verified in AD and found to contribute to memory impairment. Moreover, the authors showed that H19 knockdown or miR-129 upregulation improved cell viability and inhibited apoptosis in Aβ_25-35_-intoxicated cells [92]. Likewise, **LINC00507** has been found to be significantly upregulated in the hippocampus and cerebral cortex in mouse and cellular models of AD. Additional experiments showed that LINC00507 can bind to miR-181c-5p and antagonize its function. Therefore, LINC00507 indirectly regulates the levels of two genes involved in AD progression, *MAPT* (microtubule-associated protein tau), and *TTBK1* (tau-tubulin kinase-1-encoding tau kinase), and eventually leading to enhanced Tau hyperphosphorylation by activating the p25/p35/Cdk5 and GSK3β signaling pathways (Table S1) [93]. Similarly, Li et al. reported an increased level of **LoNA** (long nucleolus-specific lncRNA) and consistently decreased rRNA levels in the brain in AD mouse model and found that knockdown of hippocampal LoNA can rescue learning and memory impairment observed in AD mice. LoNA was first identified in mouse neuroblastoma cell nucleoli as an important regulator of RNA polymerase I transcription and of pre-rRNA processing and modifications. Silencing of LoNA leads to increased levels of rRNA and ribosomes and upregulated protein translation. Additionally, the transport of ribosomes to synapses is enhanced, resulting in increased expression of AMPA/NMDA receptors and flexibility of synapses, eventually improving long-term memory [94].

In addition, comprehensive hippocampal profiling using microarray analysis identified 1440 differentially expressed lincRNAs in mice with AD compared to control mice. Specific study of **E230001N04Rik** further confirmed the increased abundance of this lincRNA in AD cellular models and showed that E230001N04Rik can modulate the level of Tau by affecting the expression of its neighboring genes, *Srpk1* (serine/arginine-rich splicing factor kinase 1) and *Fkbp5* (FKBP prolyl isomerase 5), encoding for proteins involved in the production of Tau as well as its stability and aggregation, respectively. Consistent with these findings, both genes exhibit elevated levels in AD patients [95]. In another study, reanalysis of microarray data identified 50 dysregulated lncRNAs in AD brains compared to unaffected brains. Among them, 19 lincRNAs showed significant downregulation, and 9 lincRNAs exhibited upregulated expression (Table 1). A more detailed investigation identified age-specific, sex-specific, and disease stage-specific lincRNAs in AD brains [96]. Furthermore, using an RNA-seq approach, Garofalo et al. demonstrated the upregulated expression of lincRNAs **CH507-513H4.4**, **CH507-513H4.6**, and **CH507-513H4.3** in PBMCs of AD patients compared to healthy individuals [97]. However, their role in AD etiology is still unknown.

## Autoimmune and Neurodegenerative Disorder Pathomechanisms Can Relate on Common lincRNAs

As presented in the chapter above, some lincRNAs appear to be involved in different diseases; this concerns MIAT (MS, SLE, PD and AD), H19 (RA, SLE, PD, AD), lnc-DC (MS, SLE and SS), PVT1 (MS, RA, SS), MEG9 (MS and RA), lincRNA-p21 (RA and PD), UCA1 (RA and PD), and NORAD (SS and PD) (Fig. 2). Furthermore, the expression of some of them can differ in one disease depending on the sample analyzed, such as MIAT (in SLE and PD), lnc-DC (in SLE), H19 (in PD), or linc0597 (in SLE). In this chapter, we focus on five lincRNAs with deregulated expression patterns in most of the disorders described, related both to autoimmune and neurodegenerative pathomechanisms: NEAT1 (MS, RA, SLE, SS, HD, PD, AD, ALS), MALAT1 (MS, RA, SLE, PD, AD, ALS), TUG1 (MS, RA, SLE, HD, PD, AD), MEG3 (MS, RA, HD, PD, AD), and XIST (MS, RA, HD, PD, AD).

### NEAT1

NEAT1 (nuclear paraspeckle assembly transcript 1) is known to be essential for the formation of stress-responsive nuclear bodies called paraspeckles and can mediate the relocalization of transcription factors from gene promoters to paraspeckles, leading to their trapping and consequent decreases transcription and translation. As an example, when present at a high level, NEAT1 binds to the splicing factor SFPQ and promotes its relocalization from the *IL-8* promoter to paraspeckles, resulting in transcriptional activation of IL-8 [98].

Upregulation of NEAT1, and further for IL-8, was confirmed in serum from RRMS and SPMS patients as well as in astrocytes, microglia, and neurons from human brains affected by **MS** [9, 99, 100]. NEAT1 has also been reported to stimulate the secretion of several chemokines and interleukins, which in turn affect monocyte-macrophage functions and T cell differentiation [101]. In another study, overexpression of NEAT1 in peripheral blood samples from patients with RRMS was shown to be inversely correlated with age at the time of MS onset and with the duration of MS disease in female patients, suggesting that the expression of lincRNAs can be regulated by sex-determined factors, hormones, and drugs [14]. Like in MS, microarray analysis of lncRNAs revealed NEAT1 that was significantly upregulated in PBMCs and serum exosomes from patients with **rheumatoid arthritis** [26]. In study performed on serum exosomes, Liu et al. explained the role of upregulated NEAT1 in the promotion of RA progression through regulation of the miR-144-3p/ROCK2/Wnt/β-catenin axis (Table S1). Serum-derived exosomes from RA patients promoted the proliferation, migration, and differentiation of CD4 + T cells into Th17 cells while inhibiting their apoptosis. NEAT1 acts as a molecular sponge of miR-144-3p and in turn promotes the expression of the miR-144-3p target *ROCK2* (Rho-associated protein kinase 2). ROCK2 is involved in immune defenses and inflammation by activating the Wnt/β-catenin pathway, which plays a crucial role in inflammation in RA. Further experiments, including studies in a mouse model of RA, demonstrated that knocking down NEAT1 significantly alleviated the clinical symptoms [102]. NEAT1 expression was also found to be upregulated in STs from patients with RA [103, 104]. Upregulated NEAT1 interacts with the histone acetyltransferase p300, thus regulating histone acetylation in the *IL-18* promoter region and inducing the expression of IL-18 [103]. Moreover, NEAT1 was shown to regulate proliferation, apoptosis, and the secretion of the inflammatory cytokines IL-1β and IL-6 via another pathway by sponging miR-204-5p (Table S1) [104].

In **systemic lupus erythematosus**, Zhang et al. reported increased expression of NEAT1 in 29 samples of PBMCs and 10 samples of monocytes from patients with SLE compared with 40 PBMC and 10 monocyte samples from healthy individuals [105]. Importantly, in SLE patients, the NEAT1 level was positively correlated with both disease activity and elevated expression of several proinflammatory cytokines, chemokines, and IFN-I response genes. Furthermore, scientists found that NEAT1 regulates the expression of proinflammatory factors through the MAPK pathway [105]. Moreover, similar to the findings in MS, a role of NEAT1 in the Th1/Th2 imbalance was recently described in SLE [106, 107]. PBMCs from 97 SLE patients and 50 healthy volunteers were studied, and higher expression of NEAT1 and a lower Th1/Th2 ratio were observed in the SLE group, which reflected upregulation of IL4 and downregulation of IFNγ [106]. Recently, scientists discovered a mechanism by which NEAT1 can promote the production of Th2-related cytokines in SLE, showing that NEAT1 inhibits the ubiquitination and thereby enhances the expression of a key protein activating Th2 cells, STAT6 [108]. In addition, in study of moDCs from female SLE patients, NEAT1 was also highly upregulated, and its expression was positively correlated with the SLEDAI score. In this study, the abundance of NEAT1 was found to be related to increased expression of a group of chemokines and inflammatory cytokines including IL-6, IL-8, and CXCL10 (C-X-C motif chemokine 10). The coexpression network constructed with the lincRNAs and mRNAs differentially expressed in moDCs of SLE patients suggested their implication in the processes of cell migration and chemokine activity and in the cytokine–cytokine receptor interaction pathway [52]. NEAT1 expression was also analyzed in PBMCs derived from 20 female patients with **Sjögren’s syndrome** compared to 10 healthy controls and was increased mainly in peripheral T cells, CD4 + T cells, and CD8 + T cells. Its expression was positively correlated with the course of the disease and the levels of rheumatoid factor and IgA in the serum. Furthermore, scientists have revealed that NEAT1 regulates the activation of the MAPK pathway by selective activation of the phosphorylation of p38 and ERK1/2 and, in this way, might positively affect NEAT-induced inflammatory factors such as CXCL8 and TNF-α. Enhanced expression of both of these factors has been reported in the salivary glands of SS patients and in animal models of SS, supporting their role in the pathogenesis of this disease [109].

NEAT1, an essential component of paraspeckles, is highly abundant and ubiquitously expressed and plays an important role in the CNS; therefore, its deregulation is implicated in neurodegenerative diseases. And thus, upregulation of NEAT1 expression was reported by reanalysis of microarray data from human **Huntington’s disease** brains [69]. Similarly, Chanda et al. reported NEAT1 upregulation in HD mouse brains and cellular models. Upregulation of NEAT1 expression leads to increased formation of mHTT aggregates and an elevated level of endogenous p53 [110]. An elevated level of NEAT1_1 (short isoform) has been further confirmed in postmortem human HD brains and R6/2 mouse brains [111]. In contrast, Sunwoo et al. reported that overexpression of NEAT1_1 RNA in neuro2A cells decreased the rate of cell death during oxidative stress, suggesting a cell-protective role of NEAT1 in HD pathogenesis [111]. Moreover, Cheng et al. reported upregulation of NEAT1_2 (long isoform) in striatal neurons of HD patients and in a mouse model. NEAT1_2 upregulation was found to be mHTT-dependent, as mHTT knockdown restored the normal level of NEAT1_2 both in vitro and in vivo. By modulating the level of NEAT1_2 in cells, Cheng et al. also provided further evidence supporting the neuroprotective effect of NEAT1 against mHTT-induced cytotoxicity [112].

Several studies have reported upregulation of NEAT1 in cellular and mouse models of **Parkinson’s disease** as well as in the PB and SN of PD patients [113–119]. NEAT1 can decrease cell viability and promote apoptosis and neuroinflammation through mechanisms including upregulation of proinflammatory cytokines, including IL-1β, IL-6, and TNF-α [113–119]. NEAT1 expression was shown to be positively correlated with α-syn expression in the brain tissues of PD mice and in a cellular model [119]. Sun and coworkers found that NEAT1 promotes α-syn-induced activation of the NLRP3 (NLR family pyrin domain containing 3) inflammasome in MPP^+^-induced SH-SY5Y cells [119]. Recently, NEAT1 was described as an important component of the ceRNA network, antagonizing miRNA function and therefore regulating pathological processes in PD. NEAT1 can sequester neuroprotective miR-124 [119] and miR-374c-5p [113] (Table S1). It has also been reported to modulate six other regulatory axes: miR-1277-5p/ARHGAP26 (Rho GTPase activating protein 26), miR-212-3p/AXIN1 (axis inhibition protein 1), miR-1301-3p/GJB1 (gap junction protein beta 1), miR-124-3p/PDE4B (phosphodiesterase 4B), miR-212-5p/RAB3IP (RAB3A-interacting protein), and miR-519a-3p/SP1 (specific protein 1) (Table S1). ARHGAP26, AXIN1, GJB1, RAB3IP, and SP1 have already been associated with Parkinson’s disease, and PDE4B is known to regulate neuroinflammation [114–119]. NEAT1 has also been shown to promote MPP^+^-induced autophagy, as shown by the increased LC3-II/LC3-I (microtubule-associated proteins 1A/1B light chain 3B) ratio in the midbrain of PD mice and in a cellular model of PD. Mechanistically, this lincRNA is involved in stabilization of the mitochondrial kinase PINK1, encoded by a PD-related susceptibility gene associated with mitochondrial dysfunction and autophagy [119]. In summary, all aforementioned studies indicate the detrimental role of NEAT1 upregulation in neuronal injury and the progression of PD. However, these findings have been challenged by Simchovitz and coworkers, who suggested a protective role for NEAT1 in PD. These authors demonstrated that upregulation of NEAT1 in the SN of PD patients and in cellular models of oxidative stress is correlated with increased nuclear paraspeckle formation in dying SN neurons and is a hallmark of a neurodegenerative process. NEAT1 was found to increase cell viability and induce the formation of paraspeckles in HEK293T and SH-SY5Y cells treated with oxidative stress agents. Furthermore, these authors proposed that NEAT1 can act as a natural inhibitor of LRRK2 because by sequestering this protein in paraspeckles, it can protect cells against LRRK2-mediated damage occurring under oxidative stress conditions and in PD dopaminergic neurons [119].

Significant upregulation of NEAT1 has also been reported in the temporal cortex and hippocampus of patients with **Alzheimer’s disease**, in rat and mouse models of AD, and in cellular models of AD [120–124]. A strong positive correlation was observed between the expression levels of NEAT1 and CDK5R1 (cyclin-dependent kinase 5 regulatory subunit 1), which is known to be associated with AD development [120]. Further studies in cellular models of AD revealed that NEAT1 exacerbates Aβ-induced neuronal injury by increasing the level of pTau and sponging miR-107. Consistent with these findings, miR-107 was found to be downregulated in AD brains. miR-107 is known to prevent Aβ-induced impairment of the blood–brain barrier and dysfunction of endothelial cells in AD by targeting endophilin-1 mRNA (Table S1) [123]. Furthermore, by acting as a sponge for miR-124, NEAT1 can indirectly regulate the expression of BACE1 and contribute to Aβ-induced apoptosis (Table S1) [121]. Recently, it has been demonstrated that NEAT1 can regulate AD development by sponging miR-27a-3p (Table S1) [124]. Another study provided evidence that NEAT1 can interact with the E3 ubiquitin ligase NEDD4L and promote the ubiquitination and degradation of PINK1, leading to impairment of PINK1-mediated autophagy [122].

### MALAT1

MALAT1 (metastasis-associated lung adenocarcinoma transcript 1) is a highly conserved, ubiquitously expressed nuclear retained lincRNA that specifically localizes to nuclear domains known as nuclear speckles. It is broadly recognized as a predictive marker of metastasis in several types of cancers [125]. MALAT1 regulates the expression of a variety of genes by affecting the transcription, splicing, and formation of ribonucleoprotein complexes [126, 127].

Moreover, MALAT1 involvement in inducing the anti-inflammatory response has been described. Reduced MALAT1 levels in macrophages lead to their polarization into the proinflammatory M1 phenotype and the production of inflammatory cytokines. In CD4 + T cells, a reduction in MALAT1 expression stimulates T cell differentiation into pathogenic Th1 and Th17 phenotypes [127]. In accordance with that, the MALAT1 level was decreased in serum exosomes and FLSs from patients with **rheumatoid arthritis** [26, 128]. As reported by Li et al., in healthy FLSs, MALAT1 inhibits the inflammation and cell proliferation and induces apoptosis. By recruiting methyltransferases to increase promoter methylation, MALAT1 inhibits the transcription and expression of β-catenin, a critical molecule in the Wnt signaling pathway. Silencing of MALAT1 stimulates the nucleation of β-catenin and the secretion of inflammatory cytokines, including IL-6, IL-10, and TNF-α, inducing the proliferation and inflammation of FLSs [128]. In **multiple sclerosis**, Fenoglio et al. showed downregulation of MALAT1 in studies of PBMC samples from 27 patients with RRMS, 13 patients with PPMS, and 31 healthy controls in the Italian population [19].

However, in 2017, based on available microarray data, Cardamone et al. reported an increased level of MALAT1 in PB cells of patients with RRMS and confirmed its role in both the expression of splicing factors (HNRNPF and HNRNPH1) and alternative splicing events in MS-associated pre-mRNAs (*IL7R* and *SP140*) [126]. Upregulation of MALAT1 was also detected in serum obtained from SPMS patients [11]. Recently, the roles of NEAT1 and MALAT1 in the Th1/Th2 imbalance in MS were also described. NEAT1 and MALAT1 exert their effects through miR-544a and miR-210-3p, respectively, both of which target the *RUNX3* gene (Table S1). RUNX3 regulates the production of IFNγ in Th1 cells and IL4 in Th2 cells via TBX21 and GATA3, respectively. Dysregulation of the levels of both these miRNAs and their downstream targets consequently leads to an IFNγ(Th1)/IL4(Th2) imbalance, which plays an important role in the regulation of inflammation in MS [107].

Similarly to MS, analysis of PBMCs, T cells, B cells, and monocytes from 36 Chinese patients with **systemic lupus erythematosus** compared with 45 healthy controls revealed that MALAT1 expression was abnormally increased in the SLE patients, with predominant overexpression in monocytes. This increase was accompanied by stimulated production of the proinflammatory cytokine IL-21 in primary monocytes and activation of the SIRT1 signaling pathway, which was shown to contribute to the initiation and maintenance of lupus [129].

MALAT1 expression is also upregulated in **PD** brains as well as in mouse and cellular models of PD [75, 130–134] and was suggested to promote neuronal apoptosis by acting as a miR-124 sponge [132]. Many studies have reported significant downregulation of miR-124 in PD models, which prevents it from playing its neuroprotective role against dopaminergic cell death, autophagy dysregulation, neuroinflammation, and oxidative damage (Table S1) [135]. Furthermore, MALAT1 can contribute to MPP^+^-induced apoptosis by regulating the miR-205-5p/LRRK2, miR-135b-5p/GPNMB (glycoprotein nonmetastatic melanoma protein B), and miR‐124‐3p/DAPK1 axes (Table S1) [130, 131, 133]. Mutations in the *LRRK2* kinase gene, described as a frequent cause of both familial and sporadic PD, result in mitochondrial dysfunction, whereas DAPK1 is involved in dopaminergic neuron degeneration in PD [130, 131, 133]. A recent study revealed that MALAT1 can also induce inflammasome activation and reactive oxygen species production in PD models through epigenetic suppression of *NRF2* (NF-E2-related factor 2) expression [134].

In contrary, by comparing 120 **Alzheimer’s disease** patients with 120 control individuals with neurological but not neurodegenerative disorders, Zhuang et al. found downregulation of MALAT1 in cerebrospinal fluid and plasma samples from AD patients, which was associated with advanced disease severity [136]. MALAT1 neuroprotective role in AD progression was further confirmed in cellular and rat models of AD. MALAT1 was shown to attenuate apoptosis, reduce inflammation, and promote neurite outgrowth [137, 138]. Additionally, it was shown to decrease neuronal injury in the rat hippocampus [138]. Further studies revealed that MALAT1 negatively regulates the level of neurotoxic miR-125b, whose overexpression in AD patients increases neuronal apoptosis and Tau phosphorylation by activating CDK5 and p35/25. A direct target of miR-125b, FOXQ1, is involved in this effect (Table S1) [137]. Moreover, Li et al. demonstrated that MALAT1 may exert its neuroprotective effects against AD pathology by acting as a molecular sponge for miR-30b, thereby leading to upregulated expression of its target CNR1 (cannabinoid receptor 1) (Table S1). MALAT1 has also been linked to the activation of the PI3K/AKT (phosphatidylinositol 3-kinase and serine/threonine protein kinase B) signaling pathway, as overexpression of MALAT1 or CNR1 in AD model cells resulted in increased phosphorylation of PIK3 and AKT [138]. As PI3K/AKT activation promotes cell survival and growth, this signaling cascade has been proposed to serve as a neuroprotective factor by preventing apoptosis and neuroinflammation by regulating downstream effector molecules [139].

Interestingly, both MALAT1 and NEAT1 are also deregulated in **amyotrophic lateral sclerosis** (ALS), which is a fatal neurodegenerative disorder characterized by progressive degeneration of motor neurons in the spinal cord, brainstem, and motor cortex. ALS leads to paralysis, loss of speech, inability to swallow, and, ultimately, death from respiratory failure. Most ALS cases are sporadic (sALS), and only approximately 5–10% of cases have a genetic background and are related to mutations in more than 20 genes, including the genes encoding SOD1 (superoxide dismutase 1), the RNA/DNA-binding proteins TDP-43 (TAR DNA-binding protein 43) and FUS/TLS (fused in sarcoma/translocated in liposarcoma), and C9orf72 (chromosome 9 open reading frame 72). The pathological hallmark of ALS is the presence of neurotoxic cytoplasmic aggregates, including proteins and RNAs, in motor neurons and glial cells, predominantly in the spinal cord [140].

The role of lincRNAs in ALS etiology was confirmed by high-throughput RNA-seq analysis of PBMCs isolated from ALS patients compared to healthy controls, which led to identification of 293 significantly deregulated lncRNAs, including 81 lincRNAs [97]. Moreover, Liu et al. utilized available RNA-seq data from blood samples obtained from 7 patients with ALS and 4 controls to construct lncRNA-miRNA-mRNA regulatory networks. MALAT1 was determined to mediate the expression of 75 genes in ALS samples by acting as a miRNA sponge and potentially regulating genes related to ALS. GO analysis of MALAT1-regulated genes showed enrichment of numerous GO terms related to ALS, for example, protein targeting to the vacuole, proteins involved in vesicle organization, and NF-κB signaling pathway. Further protein–protein interaction analyses revealed that the MALAT1-regulated module containing five genes (*SYNRG*, *ITSN2*, *AAK1*, *PICALM*, and *AP3B1)* was associated with autophagy and may play a role in ALS pathogenesis [141]*.*

The second lincRNA linked to ALS, NEAT1_2, directly interacts with ALS-associated TDP-43 and FUS/TLS [142]. NEAT1_2 was found to be highly expressed and colocalized with both proteins in paraspeckles in spinal motor neurons of patients in the early stage of sALS [143]. NEAT1_2 was found to be significantly upregulated in spinal ventral horn neurons and glial cells of patients with sALS and fALS with TDP-43 pathology [144]. Further studies revealed that the endogenously expressed mutant FUS can upregulate the expression of both NEAT1_1 and NEAT1_2 and promote excessive formation of structurally and functionally compromised paraspeckles, which, together with the observed accumulation of nonparaspeckle NEAT1_1, may contribute to disease severity in patients with ALS-FUS [145, 146]. Recently, an increased cortical level of NEAT1_1 in mice with neuronal overexpression of TDP-43 was reported. Further overexpression studies revealed that NEAT1 can function as a suppressor of TDP-43 toxicity in *Drosophila* and yeast models of TDP-43 proteinopathy [147].

### TUG1

TUG1 (taurine upregulated 1) is a spliced and polyadenylated RNA first discovered in a genomic screen of taurine-treated mouse retinal cells. Its abnormal expression has been reported in various cancer types pointing the carcinogenic role of TUG1 in promoting tumor cell proliferation and invasion [148]. Moreover, expression of the lincRNA TUG1 has been documented in the developing retina and brain, with the highest level in the cortex. TUG1 is upregulated by p53, and by interacting with PRC2 (polycomb repressive complex 2), it plays a role in the regulation of cell cycle progression and the apoptosis pathway that is active in the early stages of classical neurodegenerative diseases [149].

In consistence, in patients with **Huntington’s disease**, TUG1 expression was found to be upregulated, potentially in response to p53 activation, which has also been reported in HD [69, 149]. Elevated levels of TUG1 were also detected in the serum of **PD** patients as well as in mouse and cellular models of PD [150, 151]. Loss-of-function studies revealed that TUG1 can promote apoptosis, oxidative stress, neuroinflammation, and pathological damage. Moreover, TUG1 was shown to interact with miR-152-3p and regulate its level, consistent with the decreased expression of this miRNA in PD models. MiR-152-3p regulates the expression of the proapoptotic phosphatase PTEN (phosphatase and tensin homolog), which was found to be upregulated in both cellular and mouse models of PD (Table S1) [151] and has already been linked to neurodegenerative processes in PD [152]. In addition, TUG1 was reported to be significantly upregulated in cellular and mouse models of **Alzheimer’s disease**, and further studies correlated elevated TUG1 levels with worsened memory and spatial learning abilities, increased pathological injury and apoptosis, and decreased antioxidant capacity of hippocampal neurons in AD mice [153]. Furthermore, TUG1 was found to act as a sponge for miR-15a, which targets *ROCK1* mRNA, encoding a protein involved in the production of Aβ (Table S1). In line with that finding, the authors reported an elevated level of ROCK1 and a decreased level of miR-15a in AD mice and Aβ_25-35_-treated hippocampal neurons [153].

TUG1 level is also significantly upregulated in **rheumatoid arthritis**, as has been revealed by microarray analysis of lncRNAs in PBMCs and serum exosomes from patients [26]. Higher TUG1 expression was next confirmed in cultured primary normal human FLSs and RA FLSs and was linked to increased invasion, migration, and glucose metabolism and decreased apoptosis in RA. Furthermore, scientists have described the TUG1/miR-34a-5p/LDHA (lactate dehydrogenase A) axis, via which RA FLSs, which display an increased glucose consumption rate, can upregulate glucose metabolism due to increased expression of the glucose metabolism enzyme LDHA (Table S1) [154]. Likewise, TUG1 level was found to be upregulated in serum and peripheral blood obtained from patients with different subtypes of **multiple sclerosis** [9, 10, 14]. By further in silico prediction, TUG1 has been supposed to bind miR-20a-5p, which level was decreased in blood samples from MS patients resulting in the induction of inflammatory response (Table S1) [10]. Similar to NEAT1, the overexpression of TUG1 detected in peripheral blood samples was inversely correlated with age at the time of MS onset and with the duration of MS disease in female patients [14]. Intriguingly, studying PBMCs samples from 27 patients with RRMS, 13 patients with PPMS, and 31 healthy controls in the Italian population, Fenoglio et al. showed downregulation of TUG1 and identified a correlation between the TUG1 level and the EDSS score of the disease [19]. The decreased expression of TUG1 was further confirmed in samples from a Belgian cohort composed of 17 RRMS and 7 PPMS patients and 23 healthy controls [19]. The discrepancy between these studies and the findings in previous reports [9, 14] is explained by the different biological samples used for analysis, the different phases of the disease, and the pharmacological treatments administered. TUG1 expression was also found to be markedly lower in PBMCs from **systemic lupus erythematosus** patients and to be further decreased in SLE patients with LN, with the lower expression in monocytes than in either T or B cells. The decreased level of TUG1 was negatively correlated with the SLEDAI score, ESR (erythrocyte sedimentation rate), and disease duration, and was further suggested to be correlated with deregulation of the NF-κB signaling pathway, which ultimately leads to the development of SLE [155].

### MEG3

*MEG3* is an imprinted gene located in the DLK1-MEG3 locus present on chromosome 14q32.3 in humans, which encodes an alternatively spliced long noncoding RNA of ~ 1.7 kb that functions as tumor suppressor; its expression is lost in various human cancer cell lines [156]. In human body, MEG3 is expressed in several tissues, with the highest expression levels in the pituitary gland and different regions of the human brain. It has been documented that decreased level of MEG3 underlies the pathomechanism of several immunological and neurodegenerative diseases. MEG3 deregulation has previously been reported to occur during the development of CD4 + T cells in immune thrombocytopenic purpura, resulting in an immune imbalance of Treg and Th17 cells [157]. MEG3 has also been shown to regulate ischemic neuronal death in cerebral ischemic stroke [157, 158].

In **multiple sclerosis**, analyses of blood samples from RRMS patients revealed downregulated expression of MEG3 as well, and its level was negatively correlated with the EDSS score and with the level of its putative target, NLRC5, which stimulates the production of inflammatory cytokines [32, 158]. Increased level of NLRC5 and reduced level of MEG3 were also reported in complete Freund’s adjuvant (CFA)-induced synovial tissues and in FLSs from a rat model of **RA** [33]. Like in case of lincRNA FER1L4, hypermethylation of the *MEG3* promoter led to a reduced level of MEG3 in RA. Hypermethylation of both the *FER1L4* and *MEG3* promoters can be inhibited by the methylation inhibitor 5-azadC (5-aza-2-deoxycytidine). Thus, the expression of NLRC5 was found to be significantly decreased after 5-azadC treatment [31, 33].

MEG3 contains an NRSE upstream of its TSS (transcription start site) and concordantly was found to be downregulated in brain tissues of **HD** patients [69]. Opposite results have been obtained in cell and mouse models of HD, in which MEG3 expression was significantly increased. In this study, simultaneous knockdown of MEG3 and exogenous expression of N-terminal HTT with 83 glutamines resulted in reduced formation of mHTT aggregates and decreased expression of the p53 transcription factor, which is known to be bound and stabilized by MEG3 [110]. Moreover, the documented role of MEG3 as the PRC2 interactor and its functions in the cAMP-dependent signaling pathway and cell proliferation strongly support its involvement in the massive transcriptional abnormalities observed in HD [68]. MEG3 expression was also found to be decreased in the plasma of **PD** patients and in a cellular model, and this downregulation is related to the aggravation of nonmotor symptoms, cognitive decline, and PD stage [159]. Scientists have found that MEG3 can affect the expression of LRRK2 and that MEG3 overexpression increases viability and suppresses apoptosis in SH-SY5Y cells treated with MPP ^+^ [160]. Moreover, Yi et al. reported a decreased level of MEG3 in hippocampal tissues in a rat model of **AD** established by microinjection of Aβ_25‐35_ into the right ventricle. MEG3 was found to be capable of improving cognitive impairment, mitigating neuronal damage, decreasing the expression of Aβ_25‐35_, and suppressing astrocyte activation in hippocampal tissues through activation of the PI3K/AKT signaling pathway, which keeps the Tau protein in the hypophosphorylated state [161].

### XIST

XIST (X-inactive specific transcript) is a 15–17 kb long capped, spliced, and polyadenylated lincRNA which is retained in the nucleus and plays a crucial role for X-chromosome inactivation in female placental mammals [162].

The level of XIST expression was found to be downregulated in PBMC of patients with RR and PP subtypes of **multiple sclerosis** [19]. In contrary, the expression of XIST in cartilage tissues collected from 39 patients with **rheumatoid arthritis** was found to be upregulated, similarly to the level of STAT3 but in opposite to the level of let-7c-5p. Let-7c-5c acts as a tumor suppressor in human cancers by affecting the proliferation and apoptosis of cancer cells and inhibiting STAT3 expression, which was previously shown to be associated with the proliferation and differentiation of osteoblasts in a rat model of RA. Further analysis of this study in the rat model revealed that XIST sponges let-7c-5p, thereby upregulating STAT3 (Table S1). Both silencing of XIST or upregulation of let-7c-5p promoted the expression of osteogenic genes, inhibited inflammatory responses in osteoblasts, elevated the type I collagen protein level, and reduced the degree of cartilage tissue damage in RA rats [163].

Also in neurodegenerative disorders, HD, PD, and AD, scientists found elevated level of XIST, both in mouse and cellular models [110, 164, 165]. Although the role of this lincRNA in **HD** pathogenesis is still unknown [110], in **PD** cells XIST was shown to upregulate Sp1 (synphilin-1) by sponging miR-199a-3p (Table S1). Sp1 positively modulates the transcription and translation of LRRK2. Elevated expression of Sp1 was found to contribute to inhibition of cell proliferation and enhancement of apoptosis and to increase disease severity in a mouse model of PD [164]. In case of **AD**, Yue et al. demonstrated that XIST expression was positively correlated with the expression of Aβ_1–42_ and BACE1 but negatively correlated with that of miR-124, which is a known BACE1 regulator. Further studies confirmed that XIST exhibits a stimulatory effect on cell damage by regulating the miR‐124/BACE1 axis (Table S1) [165]. Increased expression of XIST was also confirmed in hippocampal neurons treated with Aβ_25-35_. In these cells, XIST was found to enhance Aβ_25-35_-induced toxicity, oxidative stress, and apoptosis by binding and downregulating the expression of neuroprotective miR-132, which has already been reported to be downregulated in human AD brains and involved in AD pathogenesis (Table S1) [166].

## Conclusions

The last decade has resulted in an extensive body of literature concerning lincRNAs and their roles in the pathogenesis of human diseases. Whenever possible, studies were carried out on samples derived from patients compared to healthy controls matched for age and sex. These studies identified altered expression of lincRNAs that were further linked to disrupted molecular pathways and affected the expression of proteins with important biological functions. However, the consequences were not always clear. In fact, some important issues remain to be considered, such as the number of samples used for lincRNA analysis, which was sometimes limited, and the observation that the expression levels of lincRNAs are influenced by several factors. Therefore, the biological sample chosen for the analysis, the pharmacological treatment, the stage of the disease, and the age and sex of the patients can result in different observations, as seen for the TUG1 level in MS, which was found to be high in serum and PB but low in PBMC, and for LINC00507, which exhibited decreased expression in AD brains but an increased abundance in mouse and cellular models of AD (Table 1). Despite these inconsistencies, lincRNAs are undoubtedly important regulators of the differentiation and activation of nervous and immune cells, and their manipulation could have a beneficial therapeutic effect in human diseases. Additionally, due to their differential expression in a particular disease, lincRNAs can serve as valuable biomarkers for diagnosis and monitoring disease progression. Since the function of many lincRNAs in human diseases remains unknown, additional research on lincRNAs is essential for a better understanding of autoimmune and neurodegenerative disorders. With knowledge of these functions and the molecular pathways involved, we can identify promising therapeutic targets and shed light on the treatment of these diseases.

## Supplementary Information

Below is the link to the electronic supplementary material.

Supplementary file1 (PDF 185 KB)

## Data Availability

No datasets were generated during the current study.
