# Supplementary material for: Long Intergenic Noncoding RNAs Affect Biological Pathways Underlying Autoimmune and Neurodegenerative Disorders
Source: Mol Neurobiol. 2022 Jul 7;59(9):5785–808. doi: 10.1007/s12035-022-02941-0 (PMC9395482; doi:10.1007/s12035-022-02941-0)
Supplement: Supplementary file 1 — Supplementary file1 (PDF 185 KB) [file 12035_2022_2941_MOESM1_ESM.pdf]

Title: Long intergenic noncoding RNAs affect biological pathways underlying autoimmune and neurodegenerative disorders
Journal name: Molecular Neurobiology

Patrycja Plewka[1,2], Katarzyna Dorota Raczynska[1,2]

[1]Department of Gene Expression, Institute of Molecular Biology and Biotechnology, Faculty of Biology, Adam Mickiewicz University, Poznan, Poland
[2]Center for Advanced Technology, Adam Mickiewicz University, Poznan, Poland

Corresponding author
Email: doracz@amu.edu.pl

Table S1. lincRNA/miRNA/mRNA axes involved in the pathogenesis of autoimmune and neurodegenerative diseases.

**lincRNA**	**miRNA**	**Target**	**Function/molecular pathway**	**References**
RP11-29G8.3 ↑	miR-200a, miR-141, miR-24-3p, miR-15a and miR-15b ↓	Inflammatory response genes ↑	Inflammatory response in MS	[1]
PVT1 ↓	miR-200a ↑	SMAD2, GATA3, FOXO3 ↓	TH17 cell differentiation in MS	[2–4]
PVT1 ↑	miR-145-5p ↓	-	Cell proliferation, apoptosis, inflammation by regulation of IL-1β and IL-6 level in RA	[5]
GAPLINC ↑	miR-382-5p, miR-575 ↓	-	-	[6]
PICSAR ↑	miR-4701-5p ↓		Cell proliferation, migration and invasion in RA	[7]
MIAT ↑	miR-222 ↓	CFHR5 ↑	Regulation of the level of anti-dsDNA antibody and complement component C3b in SLE	[8]
	miR-221-3p ↓	TGFBR1 ↑	Apoptosis, cell viability, inflammation and oxidative stress in PD	[9]
MIAT ↓	miR-34-5p ↑	SYT1 ↓	Apoptosis, cell viability and Parkin and TH expression in PD<br>Neuron damage and motor performance in PD mice	[10]
	miR-132 ↑	SIRT1 ↓	Apoptosis, cell viability and oxidative stress in PD	[11]
lincRNA-p21 ↑	miR-625 ↓	TRPM2 ↑	Cell viability, apoptosis, oxidative stress and inflammation in PD	[12]
	miR-1277-5p ↓	α-synuclein ↑	Cell viability and apoptosis in PD	[13]
	miR-181 ↓	PKC-δ ↑	p53-dependent microglial activation and inflammation-induced neurotoxicity in PD	[14]
H19 ↓	miR-301b-3p ↑	HPRT1 ↓	Neuronal loss and apoptosis via HPRT1-dependent regulation of Wnt/β-catenin signaling pathway in PD	[15]
	miR-585-3p ↑	PIK3R3 ↓	Apoptosis, cell viability and proliferation in PD<br>Neuronal damage and behavioral phenotype in PD mice	[16]
H19 ↑	miR-129 ↓	HMGB1 ↑	Cell viability, (cell cycle) proliferation, apoptosis and oxidative stress in AD	[17]
NORAD ↓	miR-204-5p ↑	SLC5A3 ↓	Cell viability, apoptosis, oxidative stress and inflammation in PD	[18]
UCA1 ↑	miR-423-5p ↓	KCTD20 ↑	Apoptosis, cell viability, inflammation and oxidative stress in PD	[19]
LINC01311 ↓	miR-146a-5p ↑	-	Apoptosis, proliferation, autophagy, APP accumulation in AD	[20]
LINC00507 ↑	miR-181c-5p ↓	MAPT, TTBK1 ↑	Tau protein hyperphosphorylation through the regulation of the p25/p35/Cdk5 and GSK3β signaling pathways in AD	[21]

NEAT1 ↑	miR-544a ↓	RUNX3 ↑	Th1/Th2 balance, inflammatory response in MS	[22]
	miR-144-3p ↓	ROCK2 ↑	Inflammation by activating Wnt/β-catenin signaling pathway in RA	[23]
	miR-204-5p ↓	-	Proliferation, apoptosis and secretion of inflammatory cytokines IL-1β and IL-6 in RA	[24]
	miR-124 ↓	-	Cell viability, apoptosis and inflammation in PD	[25]
		BACE1 ↑	Apoptosis in AD	[26]
	miR-374c-5p ↓	-	Proliferation, apoptosis and autophagy in PD	[28]
	miR-1277-5p ↓	ARHGAP26 ↑	Cell viability, apoptosis, oxidative stress and inflammation in PD	[29]
	miR-212-3p ↓	AXIN1 ↑	Cell viability, apoptosis and inflammation in PD	[30]
	miR-1301-3p ↓	GJB1 ↑	Apoptosis and inflammation in PD	[25]
	miR-124-3p ↓	PDE4B ↑	Cell viability, apoptosis, inflammation in PD	[27]
	miR-212-5p ↓	RAB3IP ↑	Cell viability, apoptosis, oxidative stress and inflammation in PD	[31]
	miR-519a-3p ↓	SP1 ↑	Apoptosis, cell viability and inflammation in PD	[32]
	miR-107 ↓	Endophilin-1 ↑	Cell viability and apoptosis in AD	[33]
	miR-27a-3p ↓	-	Cell viability, apoptosis, APP, Aβ, BACE1, Tau, p-Tau and cleaved caspase abundance in AD<br>Cognitive functions in AD rats	[34]
MALAT1 ↑	miR-210-3p ↓	RUNX3 ↑	Th1/Th2 balance, regulation of inflammation in MS	[22]
	miR-124 ↓	-	Apoptosis in PD	[35]
	miR-205-5p ↓	LRRK2 ↑	Cell viability and apoptosis in PD	[36]
	miR-135b-5p ↓	GPNMB ↑	Proliferation (cell viability) and apoptosis in PD	[37]
	miR-124-3p ↓	DAPK1 ↑	Cell viability and apoptosis in PD<br>Behavioral phenotype in PD mice	[38]
MALAT1 ↓	miR-125b ↑	FOXQ1 ↓	Cell viability, apoptosis, inflammation and neurite outgrowth by regulating CDK5 and p35/25 in AD	[39]
	miR-30b ↑	CNR1 ↓	Cell viability, cell cycle, apoptosis and inflammation by regulating of PI3K/AKT signaling pathway in AD<br>Neuronal damage in AD rat hippocampus	[40]
TUG1 ↑	miR-152-3p ↓	PTEN ↑	Cell viability, apoptosis, oxidative stress and inflammation in PD<br>Neuronal damage and neuroinflammation in PD mice	[41]
	miR-15a ↓	ROCK1 ↑	Cell viability, apoptosis and oxidative stress in hippocampal neurons in AD<br>Cognitive functions and pathological injury of hippocampal tissues in AD mice	[42]
	miR-34a-5p ↓	LDHA ↑	Glucose metabolism in RA	[43]
	miR-20a-5p ↓	Inflammatory response genes ↑	Inflammatory response in MS	[1]
XIST ↑	let-7c-5p ↓	STAT3 ↑	Proliferation, differentiation, and inflammatory response in osteoblasts by regulating TNF-α, IL-2, and IL-6 levels and osteogenic- related genes in RA	[44]
	miR-199a-3p ↓	Sp1 ↑	Apoptosis, cell viability, cell cycle, and LRRK2 and α-synuclein expression in PD<br>Brain injury and cognitive functions in PD mice	[45]
	miR-124 ↓	BACE1 ↑	Apoptosis in AD	[46]
	miR-132 ↓	-	Cell viability, apoptosis and oxidative stress in AD	[47]

References

1. Santoro M, Nociti V, Lucchini M et al (2020) A pilot study of lncRNAs expression profile in serum of progressive multiple sclerosis patients. Eur Rev Med Pharmacol Sci 24:3267–3273. https://doi.org/10.26355/eurrev_202003_20694

2. Eftekharian MM, Ghafouri-Fard S, Soudyab M et al (2017) Expression Analysis of Long Non-coding RNAs in the Blood of Multiple Sclerosis Patients. J Mol Neurosci 63:333–341. https://doi.org/10.1007/s12031-017-0982-1

3. Colombo T, Farina L, Macino G, Paci P (2015) PVT1: a rising star among oncogenic long noncoding RNAs. Biomed Res Int 2015:304208. https://doi.org/10.1155/2015/304208

4. Naghavian R, Ghaedi K, Kiani-Esfahani A et al (2015) miR-141 and miR-200a, Revelation of New Possible Players in Modulation of Th17/Treg Differentiation and Pathogenesis of Multiple Sclerosis. PLoS One 10:e0124555. https://doi.org/10.1371/JOURNAL.PONE.0124555

5. Tang J, Yi S, Liu Y (2020) Long non-coding RNA PVT1 can regulate the proliferation and inflammatory responses of rheumatoid arthritis fibroblast-like synoviocytes by targeting microRNA-145-5p. Hum Cell 33:1081–1090. https://doi.org/10.1007/s13577-020-00419-6

6. Mo BY, Guo XH, Yang MR et al (2018) Long Non-Coding RNA GAPLINC Promotes Tumor-Like Biologic Behaviors of Fibroblast-Like Synoviocytes as MicroRNA Sponging in Rheumatoid Arthritis Patients. Front Immunol 9:702. https://doi.org/10.3389/fimmu.2018.00702

7. Bi X, Guo XH, Mo BY et al (2019) LncRNA PICSAR promotes cell proliferation, migration and invasion of fibroblast-like synoviocytes by sponging miRNA-4701-5p in rheumatoid arthritis. EBioMedicine 50:408–420. https://doi.org/10.1016/j.ebiom.2019.11.024

8. Zhang Y, Xie L, Lu W et al (2021) LncRNA MIAT enhances systemic lupus erythematosus by upregulating CFHR5 expression via miR-222 degradation. Cent Eur J Immunol 46:17-26. https://doi.org/10.5114/CEJI.2021.105242

9. Lang Y, Zhang H, Yu H et al (2022) Long non-coding RNA myocardial infarction-associated transcript promotes 1-Methyl-4-phenylpyridinium ion-induced neuronal inflammation and oxidative stress in Parkinson's disease through regulating microRNA-221-3p/ transforming growth factor /nuclear factor E2-related factor 2 axis. Bioengineered 13:930–940. https://doi.org/10.1080/21655979.2021.2015527

10. Shen Y, Cui X, Hu Y et al (2021) LncRNA-MIAT regulates the growth of SHSY5Y cells by regulating the miR-34-5p-SYT1 axis and exerts a neuroprotective effect in a mouse model of Parkinson's disease. Am J Transl Res 13:9993-10013.

11. Xu X, Zhang Y, Kang Y et al (2021) LncRNA MIAT Inhibits MPP+-Induced Neuronal Damage Through Regulating the miR-132/SIRT1 Axis in PC12 Cells. Neurochem Res 46:3365–3374. https://doi.org/10.1007/s11064-021-03437-4

12. Ding XM, Zhao LJ, Qiao HY et al (2019) Long non-coding RNA-p21 regulates MPP+-induced neuronal injury by targeting miR-625 and derepressing TRPM2 in SH-SY5Y cells. Chem Biol Interact 307:73–81. https://doi.org/10.1016/J.CBI.2019.04.017

13. Xu X, Zhuang C, Wu Z et al (2018) LincRNA-p21 Inhibits Cell Viability and Promotes Cell Apoptosis in Parkinson's Disease through Activating α-Synuclein Expression. Biomed Res Int 2018:8181374. https://doi.org/10.1155/2018/8181374

14. Ye Y, He X, Lu F et al (2018) A lincRNA-p21/miR-181 family feedback loop regulates microglial activation during systemic LPS- and MPTP- induced neuroinflammation. Cell Death Dis 9:803. https://doi.org/10.1038/s41419-018-0821-5

15. Jiang J, Piao X, Hu S et al (2020) LncRNA H19 diminishes dopaminergic neuron loss by mediating microRNA-301b-3p in Parkinson's disease via the HPRT1-mediated Wnt/β-catenin signaling pathway. Aging (Albany NY) 12:8820. https://doi.org/10.18632/AGING.102877

16. Zhang Y, Xia Q, Lin J (2020) LncRNA H19 Attenuates Apoptosis in MPTP-Induced Parkinson's Disease Through Regulating miR-585-3p/PIK3R3. Neurochem Res 45:1700–1710. https://doi.org/10.1007/s11064-020-03035-w

17. Zhang Y-Y, Bao H-L, Dong L-X et al (2021) Silenced lncRNA H19 and up-regulated microRNA-129 accelerates viability and restrains apoptosis of PC12 cells induced by Aβ25-35 in a cellular model of Alzheimer's disease. Cell Cycle 20:112–125. https://doi.org/10.1080/15384101.2020.1863681

18. Zhou S, Zhang D, Guo J et al (2020) Long non-coding RNA NORAD functions as a microRNA-204-5p sponge to repress the progression of Parkinson's disease in vitro by increasing the solute carrier family 5 member 3 expression. IUBMB Life 72:2045–2055. https://doi.org/10.1002/IUB.2344

19. Zheng Y, Liu J, Zhuang J et al (2021) Silencing of UCA1 Protects Against MPP+-Induced Cytotoxicity in SK-N-SH Cells via Modulating KCTD20 Expression by Sponging miR-423-5p. Neurochem Res 46:878–887. https://doi.org/10.1007/s11064-020-03214-9

20. Fan Y, Zhang J, Zhuang X et al (2021) Epigenetic transcripts of LINC01311 and hsa-miR-146a-5p regulate neural development in a cellular model of Alzheimer's disease. IUBMB Life 73:916–926. https://doi.org/10.1002/IUB.2472

21. Yan Y, Yan H, Teng Y et al (2020) Long non-coding RNA 00507/miRNA-181c-5p/TTBK1/MAPT axis regulates tau hyperphosphorylation in Alzheimer's disease. J Gene Med 22:e3268. https://doi.org/10.1002/JGM.3268

22. Azari H, Karimi E, Shekari M et al (2021) Construction of a lncRNA-miRNA-mRNA network to determine the key regulators of the Th1/Th2 imbalance in multiple sclerosis. Epigenomics 13:1797–1815. https://doi.org/10.2217/epi-2021-0296

23. Liu R, Jiang C, Li J et al (2021) Serum-derived exosomes containing NEAT1 promote the occurrence of rheumatoid arthritis through regulation of miR-144-3p/ROCK2 axis. Ther Adv Chronic Dis 12:2040622321991705. https://doi.org/10.1177/2040622321991705

24. Xiao J, Wang R, Zhou W et al (2021) LncRNA NEAT1 regulates the proliferation and production of the inflammatory cytokines in rheumatoid arthritis fibroblast-like synoviocytes by targeting miR-204-5p. Hum Cell 34:372–382. https://doi.org/10.1007/s13577-020-00461-4

25. Boros FA, Vécsei L, Klivényi P (2021) NEAT1 on the Field of Parkinson's Disease: Offense, Defense, or a Player on the Bench? J Parkinsons Dis 11:123-138. https://doi.org/10.3233/JPD-202374

26. Zhao M-Y, Wang G-Q, Wang N-N et al (2019) The long-non-coding RNA NEAT1 is a novel target for Alzheimer's disease progression via miR-124/BACE1 axis. Neurol Res 41:489–497. https://doi.org/10.1080/01616412.2018.1548747

27. Chen M-Y, Fan K, Zhao L-J et al (2021) Long non-coding RNA nuclear enriched abundant transcript 1 (NEAT1) sponges microRNA-124-3p to up-regulate phosphodiesterase 4B (PDE4B) to accelerate the progression of Parkinson’s disease. Bioengineered 12:708–719. https://doi.org/10.1080/21655979.2021.1883279

28. Dong L i, Zheng Y, Gao L, Luo X (2021) lncRNA NEAT1 prompts autophagy and apoptosis in MPTP-induced Parkinson’s disease by impairing miR-374c-5p. Acta Biochim Biophys Sin (Shanghai) 53:870–882. https://doi.org/10.1093/ABBS/GMAB055

29. Zhou S, Zhang D, Guo J et al (2021) Deficiency of NEAT1 prevented MPP+-induced inflammatory response, oxidative stress and apoptosis in dopaminergic SK-N-SH neuroblastoma cells via miR-1277-5p/ARHGAP26 axis. Brain Res 1750:147156. https://doi.org/10.1016/J.BRAINRES.2020.147156

30. Liu T, Zhang Y, Liu W, Zhao J (2020) LncRNA NEAT1 Regulates the Development of Parkinson’s Disease by Targeting AXIN1 Via Sponging miR-212-3p. Neurochem Res 46:230–240. https://doi.org/10.1007/S11064-020-03157-1

31. Liu R, Li F, Zhao W (2020) Long noncoding RNA NEAT1 knockdown inhibits MPP+-induced apoptosis, inflammation and cytotoxicity in SK-N-SH cells by regulating miR-212-5p/RAB3IP axis. Neurosci Lett 731:135060. https://doi.org/10.1016/J.NEULET.2020.135060

32. Wang S, Wen Q, Xiong B et al (2021) Long Noncoding RNA NEAT1 Knockdown Ameliorates 1-Methyl-4-Phenylpyridine–Induced Cell Injury Through MicroRNA-519a-3p/SP1 Axis in Parkinson Disease. World Neurosurg 156:e93–e103. https://doi.org/10.1016/J.WNEU.2021.08.147

33. Ke S, Yang Z, Yang F et al (2019) Long Noncoding RNA NEAT1 Aggravates Aβ-Induced Neuronal Damage by Targeting miR-107 in Alzheimer’s Disease. Yonsei Med J 60:640-650. https://doi.org/10.3349/YMJ.2019.60.7.640

34. Dong L-X, Zhang Y-Y, Bao H-L et al (2021) LncRNA NEAT1 promotes Alzheimer’s disease by down regulating micro-27a-3p. Am J Transl Res 13:8885-8896

35. Liu W, Zhang Q, Zhang J, et al (2017) Long non-coding RNA MALAT1 contributes to cell apoptosis by sponging miR-124 in Parkinson disease. Cell Biosci 7:19. https://doi.org/10.1186/S13578-017-0147-5

36. Chen Q, Huang X, Li R (2018) lncRNA MALAT1/miR-205-5p axis regulates MPP+-induced cell apoptosis in MN9D cells by directly targeting LRRK2. Am J Transl Res 10:563-572

37. Lv K, Liu Y, Zheng Y et al (2021) Long non-coding RNA MALAT1 regulates cell proliferation and apoptosis via miR-135b-5p/GPNMB axis in Parkinson’s disease cell model. Biol Res 54:10. https://doi.org/10.1186/S40659-021-00332-8

38. Lu Y, Gong Z, Jin X, et al (2020) LncRNA MALAT1 targeting miR-124-3p regulates DAPK1 expression contributes to cell apoptosis in Parkinson’s Disease. J Cell Biochem 121:4838–4848. https://doi.org/10.1002/JCB.29711

39. Ma P, Li Y, Zhang W et al (2019) Long Non-coding RNA MALAT1 Inhibits Neuron Apoptosis and Neuroinflammation While Stimulates Neurite Outgrowth and Its Correlation With MiR-125b Mediates PTGS2, CDK5 and FOXQ1 in Alzheimer’s Disease. Curr Alzheimer Res 16:596–612. https://doi.org/10.2174/1567205016666190725130134

40. Li L, Xu Y, Zhao M, Gao Z (2020) Neuro-protective roles of long non-coding RNA MALAT1 in Alzheimer's disease with the involvement of the microRNA-30b/CNR1 network and the following PI3K/AKT activation. Exp Mol Pathol 117:104545. https://doi.org/10.1016/J.YEXMP.2020.104545

41. Zhai K, Liu B, Gao L (2020) Long-Noncoding RNA TUG1 Promotes Parkinson's Disease via Modulating MiR-152-3p/PTEN Pathway. Hum Gene Ther 31:1274–1287. https://doi.org/10.1089/HUM.2020.106

42. Li X, Wang S-W, LI X-L et al (2020) Knockdown of long non-coding RNA TUG1 depresses apoptosis of hippocampal neurons in Alzheimer's disease by elevating microRNA-15a and repressing ROCK1 expression. Inflamm Res 69:897–910. https://doi.org/10.1007/S00011-020-01364-8

43. Zhang M, Lu N, Guo XY et al (2021) Influences of the lncRNA TUG1-miRNA-34a-5p network on fibroblast-like synoviocytes (FLSs) dysfunction in rheumatoid arthritis through targeting the lactate dehydrogenase A (LDHA). J Clin Lab Anal 35:e23969. https://doi.org/10.1002/jcla.23969

44. Wang ZQ, Xiu DH, Jiang JL, Liu GF (2020) Long non-coding RNA XIST binding to let-7c-5p contributes to rheumatoid arthritis through its effects on proliferation and differentiation of osteoblasts via regulation of STAT3. J Clin Lab Anal 34:e23496. https://doi.org/10.1002/jcla.23496

45. Zhou Q, Zhang MM, Liu M et al (2021) LncRNA XIST sponges miR-199a-3p to modulate the Sp1/LRRK2 signal pathway to accelerate Parkinson's disease progression. Aging (Albany NY) 13:4115. https://doi.org/10.18632/AGING.202378

46. Yue D, Guanqun G, Jingxin L et al (2020) Silencing of long noncoding RNA XIST attenuated Alzheimer's disease-related BACE1 alteration through miR-124. Cell Biol Int 44:630–636. https://doi.org/10.1002/CBIN.11263

47. Wang X, Wang C, Geng C, Zhao K (2018) LncRNA XIST knockdown attenuates Aβ25-35-induced toxicity, oxidative stress, and apoptosis in primary cultured rat hippocampal neurons by targeting miR-132. Int J Clin Exp Pathol 11:3915-3924
